# Electrochemical Hydroxylation of C_3_–C_12_*n*-Alkanes by Recombinant Alkane Hydroxylase (AlkB) and Rubredoxin-2 (AlkG) from *Pseudomonas putida* GPo1

**DOI:** 10.1038/s41598-017-08610-w

**Published:** 2017-08-21

**Authors:** Yi-Fang Tsai, Wen-I Luo, Jen-Lin Chang, Chun-Wei Chang, Huai-Chun Chuang, Ravirala Ramu, Guor-Tzo Wei, Jyh-Myng Zen, Steve S.-F. Yu

**Affiliations:** 1 Institute of Chemistry, Academia Sinica, Taipei, 115 Taiwan; 2 Department of Chemistry, National Chung Hsing University, Taichung, 402 Taiwan; 3 Department of Chemistry and Biochemistry, National Chung Cheng University, Chia-yi, 621 Taiwan

## Abstract

An unprecedented method for the efficient conversion of C_3_–C_12_ linear alkanes to their corresponding primary alcohols mediated by the membrane-bound alkane hydroxylase (AlkB) from *Pseudomonas putida* GPo1 is demonstrated. The X-ray absorption spectroscopy (XAS) studies support that electrons can be transferred from the reduced AlkG (rubredoxin-2, the redox partner of AlkB) to AlkB in a two-phase manner. Based on this observation, an approach for the electrocatalytic conversion from alkanes to alcohols mediated by AlkB using an AlkG immobilized screen-printed carbon electrode (SPCE) is developed. The framework distortion of AlkB–AlkG adduct on SPCE surface might create promiscuity toward gaseous substrates. Hence, small alkanes including propane and *n*-butane can be accommodated in the hydrophobic pocket of AlkB for C–H bond activation. The proof of concept herein advances the development of artificial C–H bond activation catalysts.

## Introduction

Artificial direct oxidation on terminal C–H bond of linear alkanes leading to primary alcohols in a highly selective manner is acknowledged to be challenging^1–5^. On the contrary, alkane monooxygenase (AlkB), an integral membrane-bound diiron ω-hydroxylase obtained from the Gram-negative bacterium *Pseudomonas putida* GPo1 (formerly known as *Pseudomonas oleovorans*) found in nature, can regio-selectively introduce molecular oxygen onto the unreactive terminal methyl group of C_5_−C_12_ linear alkanes to yield primary alcohols and water in the presence of reducing equivalents (eq. 1). Taking advantage of this protein, *P*. *putida* GPo1 is able to utilize linear medium-chain length alkanes (C_5_−C_12_) as the sole source of carbon and energy^6–9^.

1 $${{\rm{C}}}_{{\rm{n}}}{{\rm{H}}}_{\text{2n}+2}+{{\rm{O}}}_{2}\,+\,{\rm{NADH}}+{{\rm{H}}}^{+}\to {{\rm{C}}}_{{\rm{n}}}{{\rm{H}}}_{{\rm{2n}}+1}{\rm{OH}}+{{\rm{H}}}_{2}{\rm{O}}+{{\rm{NAD}}}^{+},{\rm{n}}=5-12$$

AlkB belongs to a family of integral membrane non-heme diiron proteins including integral membrane fatty acid desaturase (for instance, integral membrane stearoyl-acyl carrier protein ∆^9^-desaturase, SCD1) and xylene monooxygenase (XylM)^10^. Nine conserved histidine residues are found as their featured motif. This class of non-heme diiron proteins is distinct from soluble non-heme diiron proteins and their diiron active sites are ligated by multiple histidine residues. A comparison between soluble and membrane bound non-heme diiron proteins as well as the sequence alignment data among AlkB, SCD1 and XylM are illustrated in Supplementary Information, Tables S1 and S2 to reveal their functional features as well as structural and biochemical characteristics.

Besides AlkB, a soluble rubredoxin-2 (AlkG) and a soluble NADH-dependent rubredoxin reductase (AlkT) are also required to mediate the transfer of reducing equivalents for the activation of linear alkanes^11–17^.

**Figure 1:**
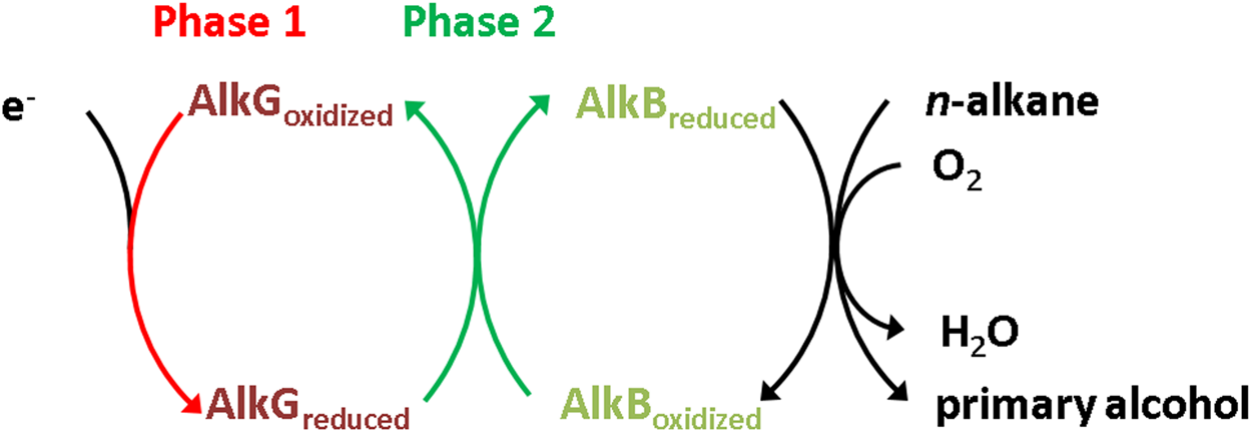
Electron pathway and protein components involved in the catalytic oxidation of medium-chain length n-alkane. The oxidation is mediated by AlkB with electrons supplied from AlkG.

However, AlkB, just like many other membrane proteins, is difficult to be isolated and purified into homogeneity, and its activity is hard to be maintained outside of the bilayer membrane^18–21^. Hence, there is a lack of detailed structural information. To date, the metal active-site, the environment around the active-site and the framework of this protein are still elusive^22^. Based on studies using diagnostic probes, it is known that a long-lived radical intermediate is generated during the catalysis mediated by AlkB^23–26^. The specific activity for the direct conversion from *n*-octane to 1-octanol is determined to be 26.6 min^−1^ ^14^; whereas the NADH consumption rate is within the range of 2.0–5.2 U/mg AlkB (one unit of activity is defined as the oxidation of 1 μmol of NADH per minute; i.e. around 90–230 min^−1^)^18–21^. To explore the structural properties and the electron transfer pathway of AlkB, X-ray absorption spectroscopy (XAS) is employed to reveal the environment close to the active site of AlkB and AlkG, respectively. Based on the information obtained, a novel electrochemical hydroxylation of C_3_–C_12_ *n*-alkanes mediated by membrane-bound AlkB through an AlkG immobilized screen-printed carbon electrode (SPCE) is developed (Fig. 1).

So far, SPCE has been extensively used for numerous purposes including analysis in evironmental assessment, food processing and pharmaceutical industry. One particular application is to serve as biosensors for the detection of biomolecues via direct electron transfer (DET)^27–29^. For instance, the detection of glucose via DET using a glucose oxidase immobilized SPCE was reported^30^. Recently developed SPCE with better operating performance significantly reduces the intrinsic barrier of DET from the electrode surface towards the redox center of proteins, that is usually hampered by the relatively thick molecular layer with poor conductivity as well as low electron transfer efficiency^31^. It is expected that DET can be conducted on a SPCE even with highly insulated membrane-bound AlkB.

This study demonstrates the electrochemically controlled redox-driven functionalization of chemically inert alkanes by AlkB on a SPCE as well as the possibility in the future chemical transformation through similar systems.

## Results

### Construction and purification of AlkB and AlkG proteins

Constructed vectors, *pET21alkBStrep* and *pACYCDuetalkG*, were generated from the insertion of *alkB* and *alkG* genes into pET-21a(+) (Novagen) and pACYCDuet-1 (Novagen), respectively. To follow the preparation of Roujeinikova, A. *et al*.^21^, a Strep II tag (Trp-Ser-His-Pro-Gln-Phe-Glu-Lys) and a linker (Ser-Ala) were added to the *C*-terminus of AlkB within the constructed vector *pET21alkbstrep*. The shuttle vectors were then transformed into *E*. *coli* BL21 (DE3) and expressed. The integrity of expressed AlkB and AlkG was demonstrated by peptide mass fingerprinting analysis of trypsin-digested protein fragments of respective proteins that appeared at 45 and 23 kDa on the SDS-PAGE.

The Strep II-tagged AlkB was purified into homogeneity via a Strep-Tactin column (GE Healthcare) in the presence of the detergent, *n*-dodecyl-*N*,*N*-dimethylamine-*N*-oxide (LDAO). The purified AlkG was obtained via a HiLoad Q Sepharose Fast Flow column (GE Healthcare) followed by a Phenyl Sepharose HP column (GE Healthcare). The UV-vis spectrum for the purified oxidized AlkG exhibits characteristic peaks at 378 and 495 nm, respectively (Figure S1(a) in Supplementary Information).

The protein content of AlkB was quantified using Enhanced Chemiluminescence (ECL) of Western blotting against Strep-tag; whereas that of AlkG was determined by UV-vis absorption at 378 and 495 nm.

Also, iron quantification conducted by inductively coupled plasma optical emission spectroscopy (ICP-OES) demonstrates that the iron content of purified AlkB and AlkG are 1.5 ± 0.4 and 1.6 ± 0.4 per molecule, respectively. The data is consistent with previous findings that AlkB is a diiron monooxygenase^18^ with an anti-ferromagnetically coupled high-spin Fe^III^ − Fe^III^ evidenced by Mössbauer studies, whereas AlkG contains two [Fe − (CysS)_4_] cores characterized by spectroscopic methods^32, 33^.

### X-ray absorption spectroscopic study of AlkB and AlkG proteins

**Figure 2:**
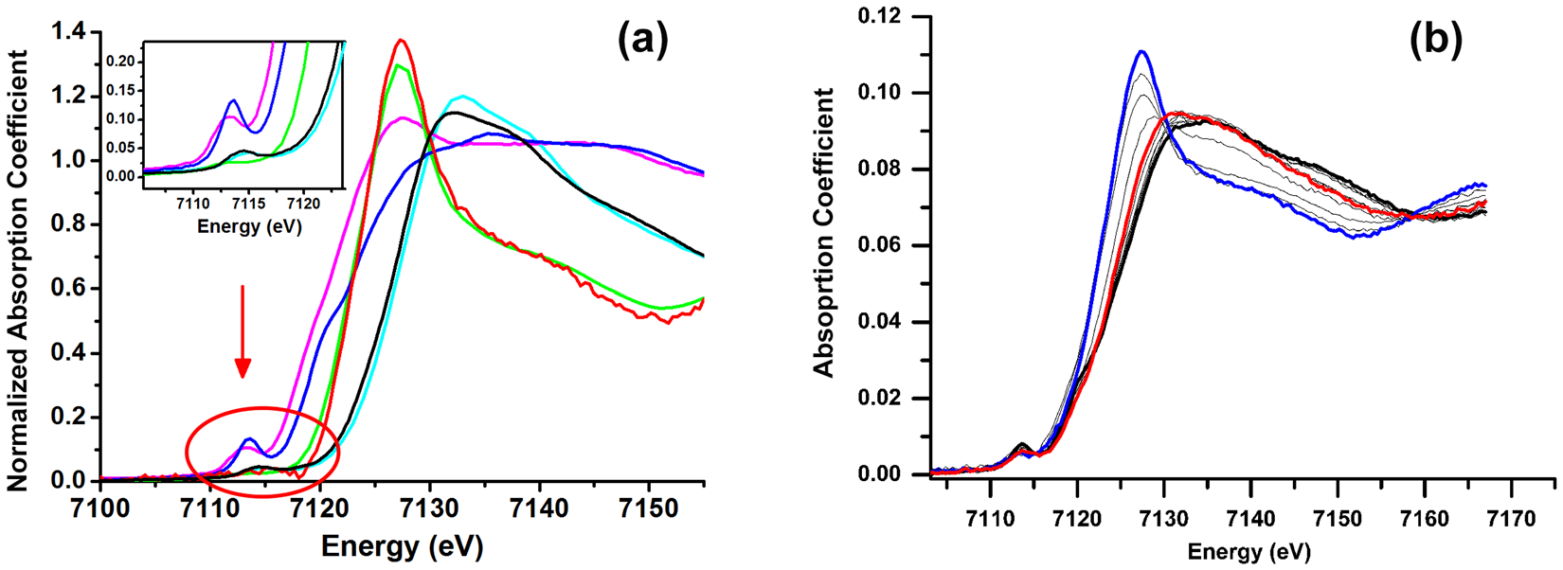
(**a**) Normalized XANES spectra of FeSO_4_ (light green), FeCl_3_ (cyan), AlkG_reduced_ (magenta), AlkG_oxidized_ (blue), AlkB_reduced_ (red) and AlkB_oxidized_ (black). The inset highlights the pre-edge absorption of AlkB_oxidized_, AlkG_oxidized_ and AlkG_reduced_ at ca. 7113 eV; (**b**) Titration of dithionite into the mixture of AlkG and AlkB with ca. 1:1 ratio (direction for the titration of dithionite, concentration from low to high: Black → Red → Blue).

Fe K-edge X-ray absorption near edge structures (XANES) of AlkB and AlKG are displayed in Fig. 2(a), providing the information of iron oxidation states and the geometry of iron atom within the proteins. Enlarged pre-edge region (inset of Fig. 2(a)) illustrates the difference in XANES among the purified AlkB (alternatively termed AlkB_oxidized_ from this point on in the text), the fully oxidized AlkG (AlkG_oxidized_) and the fully reduced AlkG (AlkG_reduced_). The mid-point energies of rising edge, defined as K-edge energies in the following text (confer Table 1), for AlkB_oxidized_ and AlkG_oxidized_ absorption appear to be 7125.7 eV and 7120.9 eV, respectively (Fig. 2(a) and Table 1). The K-edge energy of FeCl_3_ located at 7126.2 eV suggests that the purified AlkB (AlkB_oxidized_) is in the ferric state. In addition, the K-edge energies obtained from several iron-sulfur cluster containing proteins in the oxidized form, such as succicinate:caldariellaquinone oxidoreductase (SdhC)^34^, phthalate dioxygenase (PDO)^35^ and superoxide responsive protein (SoxR)^36^, are 7119.3 eV (the energy calibration is set with Fe foil at 7111.3 eV), 7120.6 eV (the energy calibration is set with Fe foil at 7111.2 eV) and 7120.2 eV, respectively. Moreover, both K-edge energies for the oxidized form of non-heme diiron hydroxylase in soluble methane monooxygenase (sMMO), MMOH_ox_, and its complex with another component in the system, MMOH_ox_ + MMOD, are 7124.7 eV (the energy calibration is set with Fe foil at 7111.1 eV)^37^. These data imply that the purified AlkB is indeed in ferric state.

**Table 1:** Results obtained from pre-edge spectra of AlkG_oxidized_, AlkG_reduced_, AlkB_oxidized_ and AlkB_reduced_.

	AlkG_oxidized_	AlkG_reduced_	AlkB_oxidized_		AlkB_reduced_
Pre-edge energy (eV)	7113.7	7112.8	7114.1	7115.9	ND^b^
Pre-edge area^a^	25.0	17.5	6.7	4.5	ND^b^
K-edge energy (eV)^c^	7120.9	7119.6	7125.7		7122.3

^a^The integrated area is reported in unit, which is the real value multiply by 100^39–41^.

^b^ND: Not detected due to the poor resolution of the spectrum after linear combination.

^c^K-edge energy^41^ (eV) is defined herein as the energy at half its maximum normalization intensity for the rising edge of normalized XANES spectra.

It has been known that AlkG can be reduced either by NADH in the presence of rubredoxin reductase, or sodium dithionite^17^. In this study, it is found that at least 20 eq. of dithionite is required to completely abolish the UV-vis feature of AlkG_oxidized_. Normalized XANES profile of AlkG_reduced_ gives the K-edge energy of 7119.6 eV (Fig. 2(a) and Table 1). This result is comparable with the K-edge energies of several iron-sulfur cluster containing proteins in the reduced form, such as PDO^35^ and SoxR^36^, which are 7118.7 eV (the energy calibration is set with Fe foil at 7111.2 eV) and 7119.3 eV, respectively.

Although it is shown that AlkG_oxidized_ can be fully reduced using a high dose of dithionite, AlkB_oxidized_ cannot be entirely reduced by dithionite (Figure S2). The treatment of AlkB_oxidized_ with 10 eq. of dithionite only slightly leads to the shift in the K-edge energy of −0.4 eV. Instead, AlkB_oxidized_ can be reduced in the presence of AlkG_reduced_. The titration of dithionite into AlkB_oxidized_–AlkG_oxidized_ mixture in 1:1 ratio was performed and the shift to lower energy in XANES profile supports that AlkB_oxidized_ can be reduced by AlkG_reduced_ (Fig. 2(b)). The XANES spectrum of AlkB in the reduced form (AlkB_reduced_) can be obtained after the subtraction of XANES spectrum of AlkG_reduced_ from that of the AlkB_reduced_–AlkG_reduced_ adduct (Fig. 2(a), red line). The obtained K-edge energy of AlkB_reduced_ is 7122.3 eV, which is comparable to that of FeSO_4_ (7122.2 eV) (Fig. 2(a) and Table 1). The K-edge energies for MMOH_red_ and the hydroxylase of toluene monooxygenase, TMOH_red_, are 7121.5 eV and 7121.8 eV (the energy calibration is set with Fe foil at 7111.1 eV for both proteins), respectively^38^, which are quite close to the obtained K-edge energy from AlkB_reduced_.

To decipher the redox chemistry occurred in between AlkB–AlkG pair, the linear combination of experimental XAS data obtained from the individual XANES profile was conducted (Figure S3 in Supplementary Information). The spectral change deduced from the linear combination suggests that two phases are involved in the electron transfer mediated by AlkB–AlkG pair. During the first phase observed through the linear combination of XANES spectra among AlkG_reduced_, AlkG_oxidized_ and AlkB_oxidized_, all AlkG_oxidized_ are fully reduced to AlkG_reduced_ prior to the reduction of AlkB_oxidized_ (Figure S3(d)). During the second phase observed through the linear combination of XANES spectra among AlkG_reduced_, AlkB_oxidized_ and AlkB_reduced_, the transition from AlkB_oxidized_ to AlkB_reduced_ in the presence of AlkG_reduced_ is noted (Figure S3(e)). The merged XANES profile from two phases (Figure S3(f)) agrees with the experimentally given XANES profile obtained from AlkB–AlkG (1:1) mixture (Fig. 2(b)). The results herein imply that AlkG serves as a redox partner to transfer electrons towards AlkB through the transition in the order of AlkB_oxidized_AlkG_oxidized_ → AlkB_oxidized_AlkG_reduced_ → AlkB_reduced_AlkG_reduced_ (Fig. 1).

**Figure 3:**
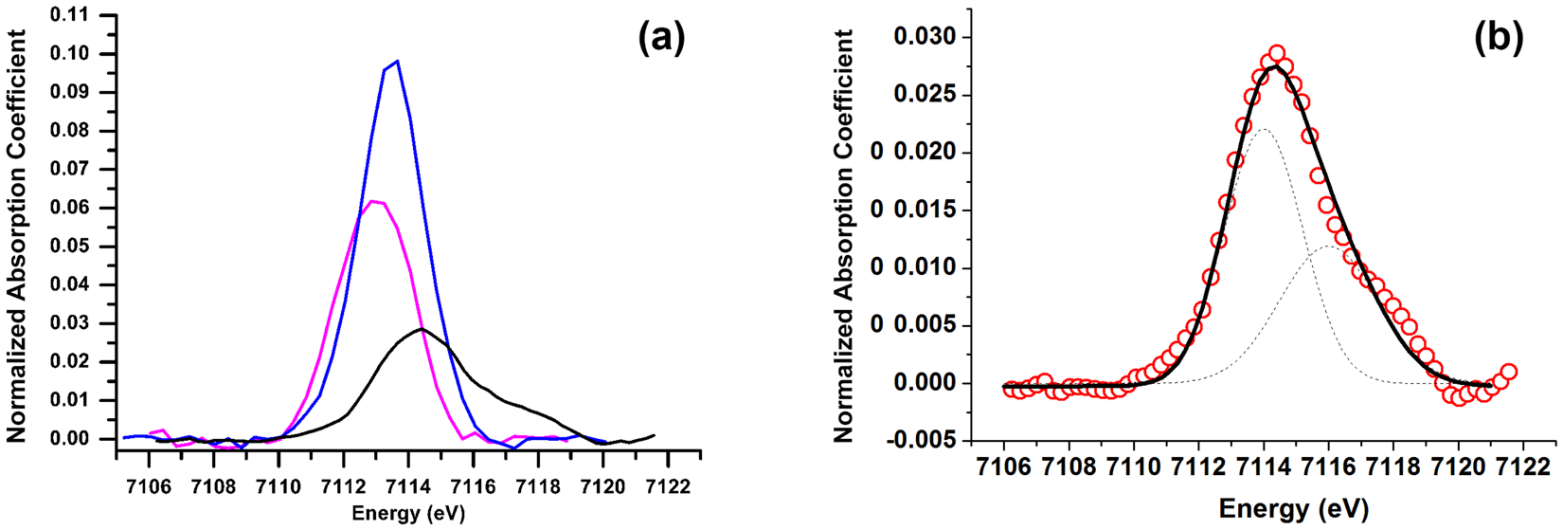
(**a**) Pre-edge spectra of AlkG_reduced_ (magenta), AlkG_oxidized_ (blue) and AlkB_oxidized_ (black) after the subtraction from background; (**b**) the pre-edge spectrum of AlkB_oxidized_ (red circled line) is de-convoluted into two pre-edge peak components (dashed lines) with the absorption maximum at 7114.1 eV and 7115.9 eV, respectively. The merged spectra of two component peaks (black line, 11.2 units) and the pre-edge spectrum of AlkB_oxidized_ are superimposable.

The strong pre-edge absorption at around 7113 eV for the iron-sulfur complex in both AlkG_oxidized_ and AlkG_reduced_ is assigned as the 1 s→3d transition, reflecting the extent of d–p mixing that satisfies the selection rule (Fig. 2(a), inset)^34^. Pre-edge absorption peaks obtained from AlkG_oxidized_ and AlkG_reduced_ were normalized and their integrated areas were calculated following an established procedure, resulting in the integrated areas for AlkG_oxdized_ and AlkG_reduced_ of 25.0 units and 17.5 units, respectively (one unit is 10^−2^ eV; Fig. 3 and Table 1)^41^. The integrated area for pre-edge absorption peaks is acknowledged to be correlated with the coordination number of iron center. Model compound studies show that, for high spin Fe^III^ model complexes, the range for the normalized integrated areas are 6–9 units, 12–19 units and 20–25 units for 6-, 5- and 4-coordinated complexes, respectively^41^. For high spin Fe^II^ model complexes, the range for the normalized integrated areas are 4–6 units, 8–13 units and 16–21 units for 6-, 5- and 4-coordinated complexes, respectively^39^. The obtained integrated areas for AlkG_oxdized_ and AlkG_reduced_ are consistent with those obtained from model complexes such as ferric (Et_4_N)[FeCl_4_] (25.0 units) and ferrous (Et_4_N)_2_[FeCl_4_] (17.4 units), where iron centres are in a non-centrosymmetric point group such as *T*_*d*_^39, 41^. The decreased integrated area of AlkG_reduced_, for ca. 70% of AlkG_oxidized_, is attributed to the higher occupancy of 3d orbitals in Fe^II^ relative to that of Fe^III^. Moreover, the difference in the K-edge energy between the oxidized state and the reduced state of AlkG is 1.3 eV (Table 1), which is smaller than the data obtained from Rieske-type clusters (ca. 2 eV)^35^ but are closer to the one electron reduction of [2Fe-2S] cluster in SoxR (0.9 eV)^36^. This result suggests that the reduction of AlkG from Fe^III^ to Fe^II^ may not dramatically affect the charge of iron centre for its coordination with cysteinyl sulfur atoms. In addition, the Fe extended X-ray absorption fine structure (EXAFS) data of AlkG merely show a minute difference between the Fe–S bond length (or Fe–S backscattering in distance) of AlkG_oxidized_ (2.25 Å) and AlkG_reduced_ (2.28 Å) (Figure S4 and Table S3 in Supplementary Information). This is consistent with the earlier EXAFS study indicating that the reduction of rubredoxin yields only a slight increase of average Fe–S bond length by ca. 0.05 Å^42^. The lack of Fe–Fe backscattering suggests that the complex possesses a tetrahedron-like Fe–4S core. Similar to common iron-sulfur proteins that participate in electron transfer, e.g. SoxR^36^, the reorganization energy for the redox switch from AlkG_oxidized_ to AlkG_reduced_ is minute, which is facile for electron transfer from AlkG to AlkB. Furthermore, the pre-edge energies (integrated intensities) for SdhC_oxidized_ and PDO_oxidized_ are 7113.0 eV (the energy calibration is set with Fe foil at 7111.3 eV; 24.8 units) and 7113.1 eV (the energy calibration is set with Fe foil at 7111.2 eV; 26.0 units), respectively; whereas that of PDO_reduced_ (Fe^II^–Fe^III^) is 7112.5 eV (18 units)^34, 35^. The obtained pre-edge energies from AlkG_oxdized_ and AlkG_reduced_ are 7113.7 eV and 7112.8 eV, respectively (Table 1), which are fairly consistent with the reported values obtained from aforementioned iron-sulfur proteins in their respective oxidation states.

The integrated pre-edge area of AlkB_oxidized_ is 11.2 units, which suggests that iron geometry in AlkB_oxidized_ is either in a 5-coordination or a distorted 6-coordination environment. The 1s→3d transition can be further resolved into two peaks at 7114.1 eV and 7115.9 eV with the integrated intensity of 6.7 and 4.5 units, respectively (Fig. 3(b)). The pre-edge profile of AlkB_oxidized_ is reminiscent of distinct pre-edge signals for diiron model complexes such as ([FeOH(H_2_O)Chel]_2_(H_2_O)_4_ and [FeOH(H_2_O)Dipic]_2_ with an intense feature at the low-energy but a shoulder at the high-energy^40^. The pre-edge profiles of MMOH_ox_ and the complex, MMOH_ox_ + MMOD, also give two resolved peaks with featured intensities of 7.0 (1.2) units and 7.5 (0.3) units at 7113.1 eV as well as 1.1 (0.1) units and 2.1 (0.7) units at 7114.6 eV, respectively^37^. The slight inconsistency both in the position and intensity of pre-edge peaks is due to the fact that, in the study of MMOH_ox_ and MMOH_ox_ + MMOD, the energy calibration is set with Fe foil at 7111.1 eV, which is 0.9 eV shorter. In addition, the normalization of XANES spectra for MMOH_ox_ and the corresponding model studies were selected at 7130 eV with an edge jump of 1. Therefore, the pre-edge intensity of AlkB_oxidized_ at around 7114.5 eV has to be lowered down by 1.1 times according to the present employed method^41^. The pre-edge intensities or areas observed for other non-heme diiron proteins such as metHrN_3_ (~10.4 units; Hr: hemerythrin), soluble stearoyl-acyl carrier protein Δ^9^–desaturase (10.8 units or 11.5 units) and RNR R2_met_ (~10.1 units; RNR R2: ribonucleotide reductase subunit R2) suggest that AlkB_oxidized_ exhibits the features of non-heme diiron proteins with the iron active sites in diferric state^43, 44^.

Similar to mammalian integral membrane stearoyl-CoA desaturase (SCD1), AlkB contains four histidine-containing motifs (two HXXHH motifs, one HXXXH motif and one NXXH motif) that presumably consist of the ligands to coordinate the bimetallic centre (see sequence alignment in Supplementary Information). Recently revealed SCD crystal structure indicates that the distance between those two metal ions (Zn) is 6.4 Å and each metal ion is associated with four to five histidine residues including the TM4 motif (N^261^XXXH^265^)^45^.

**Figure 4:**
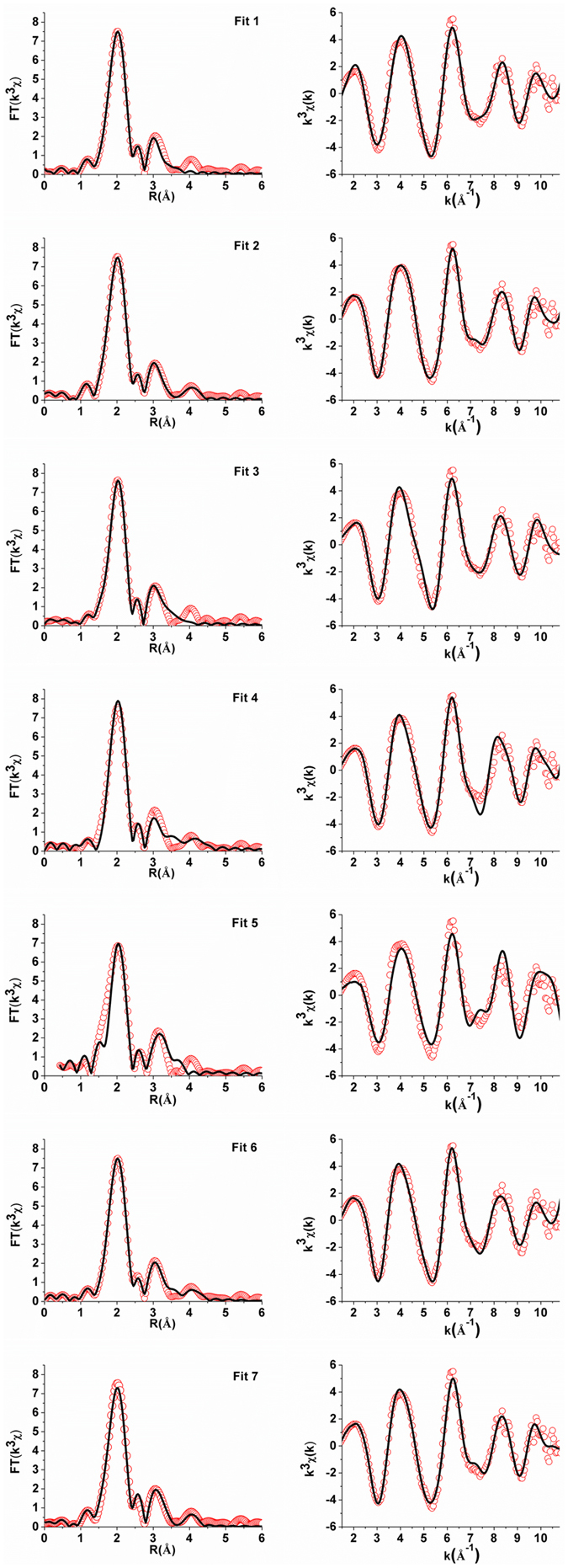
Left: Phase shift corrected Fourier transforms of Fe EXAFS (red circles) and the corresponding best fits (black solid lines); Right: Fe EXAFS (red circles) and the corresponding best fits (black solid lines) of purified AlkB.

To obtain the local structural information for the metal active site of AlkB_oxidized_, EXAFS data analysis was conducted and the resulting *k* ^3^-weighted EXAFS raw data (*k* ^3^χ, i.e. the radial distribution function), are displayed in Fig. 4. To employ the mammalian SCD crystal structure as a model system, seven possible fitting results are listed in Table 2. The first-shell data fitting (2.0 Å < R < 3.0 Å) to the Fourier transforms of *k* ^3^χ mainly arises from the backscattering from 3–5 ligated N/O atoms with the average distance of 2.02–2.03 Å. In Fit 5, a Fe–O/N of 2.40 Å with the goodness of fit, *R*_fit_, of 1.6% is considered acceptable but poorly fitted. Previous data of RNR R2_met_ show that, except for the short Fe–O’ (bridged-oxo), the average length of Fe–O/N bonds for the first coordination shell is 0.07 Å shorter than that of metHrN_3_ (2.13 Å)^46^. Since Fe–O bonds are much shorter than Fe–N bonds in high-spin ferric complexes, this result suggests that at least one histidine on the iron in metHrN_3_ is replaced by an oxyanion ligand in RNR R2_met_. The shorter Fe–O/N bonds of AlkB than that of histidine ligated metHrN_3_ (~0.10 Å shorter) is also observed for the inner shell coordination and that may be attributed to the participation of an oxyanion ligand in the coordination. Besides, previous studies demonstrate that the average Fe–O/N bond length of MMOH_ox_ or MMOH_ox_ + MMOD is within the range of 1.99–2.01 Å^37, 47^.

**Table 2:** Fitting of the *k* ^3^-weighted EXAFS data for alkane hydroxylase (AlkB).

*Fit*	Fe–O/N			Fe–O/N			Fe–C			Fe–Fe			Fe–C/N			*Fitting range*		*Goodness*-*of*-*fit*
	*N*	*R* (Å)	σ^2^ (Å^2^)	*N*	*R* (Å)	σ^2^ (Å^2^)	*N*	*R* (Å)	σ^2^ (Å^2^)	*N*	*R* (Å)	σ^2^ (Å^2^)	*N*	*R* (Å)	σ^2^ (Å^2^)	∆*k* (Å^−1^)	∆*R* (Å)	*R*_fit_ (%)
1	5	2.02	0.007							1	3.08	0.008				[1.45, 10.83]	[1.31, 3.53]	0.019
2	5	2.03	0.007				3	4.10	0.007	1	3.09	0.008				[1.38, 10.83]	[1.41, 4.20]	0.080
3	5	2.03	0.006				3	3.13	0.003							[1.35, 11.04]	[1.38, 3.52]	0.12
4	5	2.02	0.005				3	3.17	0.008	1	4.07	0.008				[1.38, 10.88]	[1.37, 4.37]	0.21
5	3	2.03	0.002	1	2.40	0.006				1	3.11	0.003				[2.49, 10.88]	[1.31, 3.53]	1.6
6	5	2.03	0.007				3	3.13	0.005				3	4.17	0.009	[1.38, 10.68]	[1.31, 4.33]	0.080
7	5	2.02	0.007				3	2.95	0.011	1	3.08	0.007	3	4.08	0.006	[1.40, 10.85]	[1.40, 4.37]	0.075

(a) Results of fits 1–7 to EXAFS data for AlkB. Errors are estimated to be 25% for coordination numbers and 0.01–0.03 Å for distances^38^. Parameters used in the fitting include: *N*, the coordination number; *R* (Å), the distance relative to Fe; *σ* ^2^ (Å^2^), the Debye-Waller factor; and *R*_fit_ (%), the goodness-of-fit parameter. (b) Fitting range for each fit. Ranges for *k* space (Å^−1^) and *R* space (Å) are indicated by Δ*k* and Δ*R*, respectively.

The second-shell data fitting to the Fourier transforms of *k* ^3^χ (3.0 Å < R < 4.0 Å) yields 1 × Fe–Fe of 3.08–3.11 Å (Fits 1, 2, 5 and 7) and 3 × Fe–C_α_ from histidine with the average distance of 2.95–3.17 Å (Fits 3, 4, 6 and 7), along with all the fitting results considered acceptable (*R*_fit_ < 2.0%). The third-shell data fitting can be extended to give 1 × Fe–C_α_ of 4.10 Å (Fit 2) or 1 × Fe–Fe of 4.07 Å (Fit 4). Since Fe^III^–Fe^III^ distance for most non-heme diiron enzymes is within the range of 3.0–3.4 Å (Table S1 in Supplementary Information), it is surmised that the active site of AlkB contains two irons with Fe^III^–Fe^III^ distance of 3.08–3.09 Å and each iron coordinates with 3–4 histidines (Fits 1, 2 and 7 in Fig. 4 and Table 2). Among Fits 1, 2 and 7, the obtained Fit 7 with the additional second- and third-shells giving 3 × Fe–C_α_ of 2.95 Å and 3 × Fe–C/N of 4.08 Å could be more promising in revealing the geometry and the structure for the iron active site of AlkB. Previous multi-shell restricted fits for non-heme diferric iron proteins, such as metHrN_3_ and RNR R2_met_, yield 1 × Fe–Fe of 3.19 Å and 3.22 Å, respectively, which is ca. 0.10 Å longer than that value obtained from AlkB^46^. In addition, previous fits for metHrN_3_ and RNR R2_met_ result in the second-shell backscatters of 3.05 Å (4.2 × Fe–C_α_) and 3.03 Å (3.1 × Fe–C_α_) as well as third-shell backscatters of 4.33 Å (5.1 × Fe–C/N) and 4.30 Å (3.8 × Fe–C/N), respectively, which are 0.09 Å (in the case of metHrN_3_) and 0.24 Å (in the case of RNR R2_met_) longer than the value obtained from Fit 7 of AlkB^46^. Based on the results that Fe–O/N bond length obtained from the first-shell fitting of AlkB is 0.03–0.10 Å shorter than that of metHrN_3_ and RNR R2_met_ (*vide supra*), the active site of AlkB seems to be with a more congested coordination environment, presumably, similar to the active site of MMOH_ox_ that with a short first-shell Fe–O/N bond (ca. 2.0 Å) and short Fe–Fe distance (ca. 3.0 Å)^37, 47^.

However, the possibility that the average distance of Fe–Fe backscatter in the purified AlkB may not be resolved by EXAFS study cannot be excluded. The third-shell data fitting in Fit 6 reveals 3 × Fe–C/N bonds with an average distance of 4.17 Å that might be resulted from the imidazole rings. Plus, in Fit 6, there is no Fe–Fe backscattering observed and this model is more consistent with the result observed from the three-dimensional structure of SCD1 with a dimetallic center of metal-metal distance >6 Å. In summary, the obtained seven Fits of EXAFS analysis (Fig. 4 and Table 2) may directly or indirectly contribute to the understanding of the iron active site in AlkB. Further studies, including single crystal X-ray crystallography, Mössbauer and/or Resonance Raman Spectroscopy, are definitely essential to unravel the three-dimensional structure and the non-heme diiron active site features of membrane-bound AlkB protein in order to comprehend how AlkB executes medium-chain length *﻿n*-alkane oxidation.

### Electrochemical properties of AlkB and AlkG proteins

XANES studies show that fully reduced AlkG can efficiently reduce AlkB towards a higher reduced state (Fig. 2(b), *vide supra*). Based on the redox sequence concluded from XANES studies, it is expected that the direct supply of electrons to AlkG_oxidized_ using an electrochemical approach followed by the shuttling of electrons to AlkB_oxidized_ can further catalytically oxidize linear alkanes to primary alcohols. Fully reduced AlkG can be prepared electrochemically through a direct electron transfer (DET) process on a disposable SPCE (with the surface roughness (Ra) of 2.02 nm) immobilized with AlkG.

It is noted that oxidized AlkG immobilized on a SPCE shows the UV-vis absorption features at 410, 495 and 595 nm (Figure S1(b) in Supplementary Information). As shown in Fig. 4(a), low charge transfer resistance (R_ct_ = 1.81 kΩ) of SPCE enables the DET from the immobilized AlkG to AlkB.

The reduction and oxidation potential of AlkG are determined to be −515 and −266 mV, respectively, in a quasi-reversible manner (Fig. 4(b)). The surface concentration of electroactive AlkG on a SPCE is estimated to be 0.057 × 10^−9^ mol/cm^2^ based on the observed cyclic voltammogram (CV)^48^.

CV of recombinant AlkB-enriched membrane obtained from *E*. *coli* without further purification gives redox peaks at −452 mV and −285 mV after deoxygenation (Fig. 4(c)). In the presence of oxygen, obvious electrocatalytic reduction is observed, indicating that AlkG and AlKB-enriched membrane immobilized on a SPCE are electrocatalytic active. The surface coverage for AlkB-enriched membrane on a SPCE is estimated to be 1.06 × 10^─9^ mol/cm^2^, which is higher than theoretical monolayer coverage (i.e. 1.89 × 10^−11^ mol/cm^2^)^49^. The Δ*E*_p,1/2_ for the redox peak of AlkB-enriched membrane is calculated as 36.9 mV, which is close to an ideal Nernstian adsorbate layer with the transfer of two electrons under Langmuir isotherm conditions (i.e. Δ*E*_p,1/2 = _45.3 mV)^50^.

Presumably, this result is attributed to the transition of two-electron transfer from Fe^III^Fe^III^ → Fe^II^Fe^II^ in the absence of substrates.

Results show that AlkG on the surface of electrodes can be maintained at the reduced state when the operation potential is kept at −0.6 V (vs. Ag/AgCl) for the catalytic turnover mediated by AlkB-enriched membrane.

It is expected that the AlkG immobilized electrode can interact with suspended AlkB-enriched membrane micelles to form an AlkB–AlkG complex and then efficiently convert C_5_–C_12_ linear alkanes to the corresponding primary alcohols.

**Figure 5:**
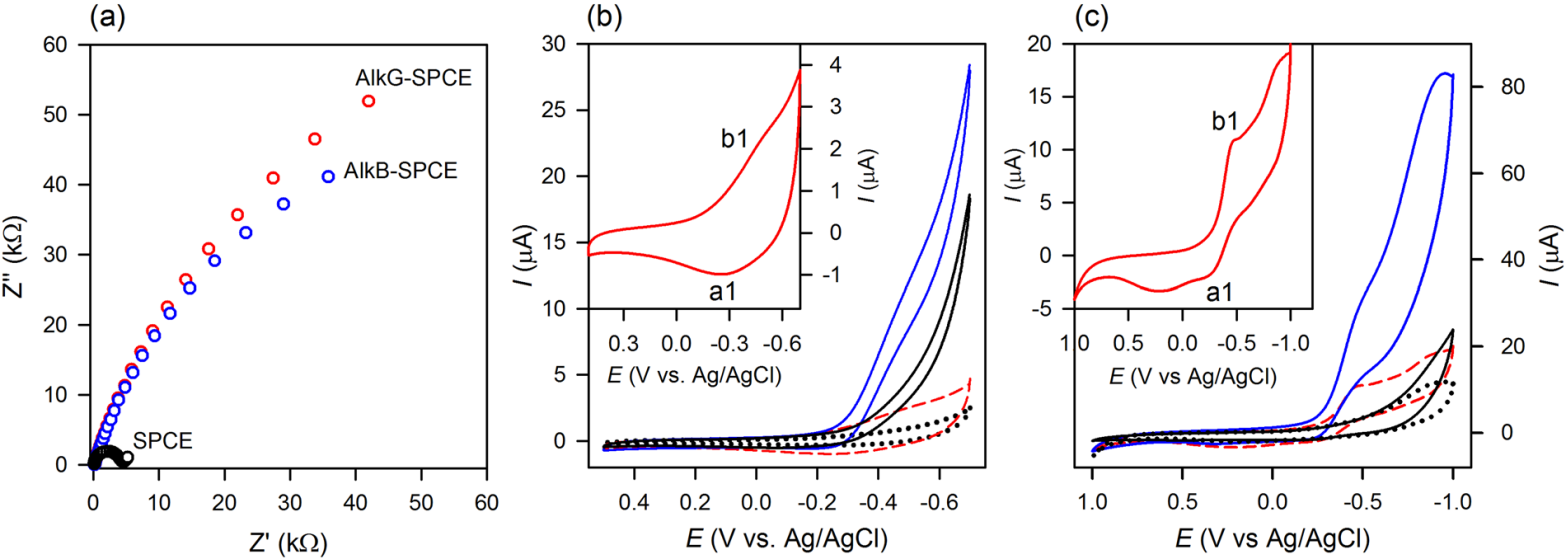
(**a**) Electrochemical impedance studies of AlkG modified SPCE, AlkB-enriched membrane SPCE, and bare SPCE in 1 mM [Fe(CN)_6_]^3−^/0.1 M KCl solution. Electrochemical studies in 0.1 M phosphate buffer solution (PBS), pH 7.4: (**b**) cyclic voltammograms (CVs) of bare SPCE measured in the absence (black dotted line) and in the presence (black solid line) of oxygen; and CVs of AlkG-SPCE measured in the absence (red dash line) and in the presence (blue solid line) of oxygen. Inset: DET of AlkG-SPCE obtained in the absence of oxygen. (**c**) CVs of bare SPCE measured in the absence (black dotted line) and in the presence (black solid line) of oxygen; and CVs of AlkB-enriched membrane SPCE in the absence (red dash line) and in the presence (blue solid line) of oxygen. Inset: DET of AlkB-enriched membrane SPCE in the absence of oxygen.

To carry out the electrochemical conversion, first, the surface of SPCE is coated with a layer of Nafion^®^. Then, an aliquot of AlkG solution (0.13 nmol/cm^2^) is immobilized on the top of electrode via physical adsorption. To optimize the conversion from *n*-octane to 1-octanol, the electrochemical reaction is suspended by varying the concentration of AlkB-enriched membrane at 43–690 μM in 50 mM Tris buffer solution, pH 7.5. No activity is observed from AlkB-enriched membrane using a SPCE without AlkG (Figure S6 in Supplementary Information). It is found that the highest specific activity can be achieved if operated within 30-min duration. The obtained specific activity in turnover frequency (TOF) per AlkB–AlkG complex (min^−1^∙protein^−1^) is related to the actual concentration of AlkB bound with the immobilized AlkG on the SPCE. A one-site binding model can fairly describe the change in the specific activity with the concentration of AlkB bound to AlkG, yielding the coefficient of determination (R^2^) of 0.984, the maximum activity of 1,100 min^−1^∙protein^−1^ as well as *K*_d_ of 268 μM (data not shown). However, if a two-site binding model is employed (Fig. 5), the change in the specific activity with the concentration of AlkB bound to AlkG of 322 and 776 min^−1^∙protein^−1^ can be obtained. Both dissociation constants, *K*_d_, from AlkB-enriched membrane are determined to be 268 μM.

### Alkane hydroxylation via electrocatalysis

**Figure 6:**
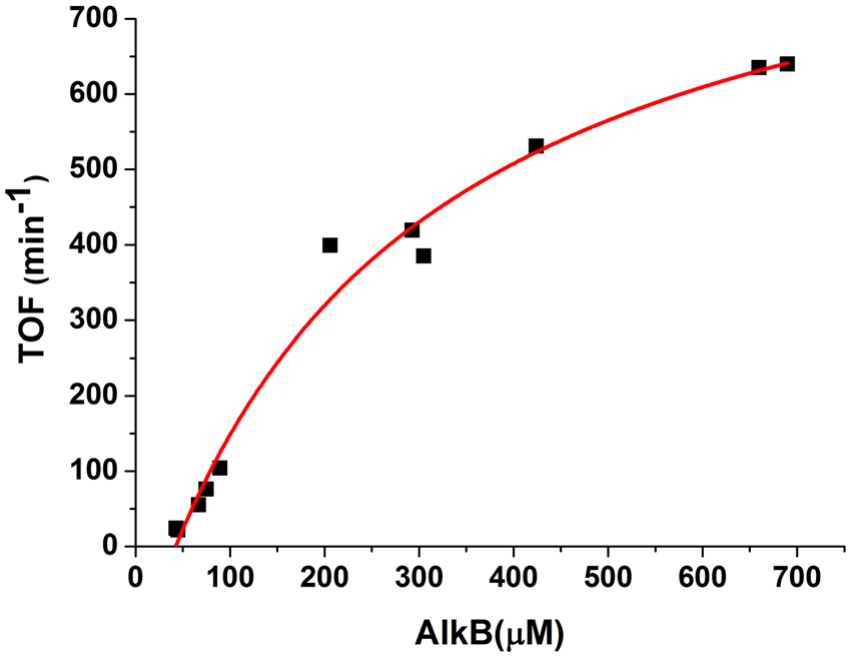
Specific activity in turnover frequency (TOF) with increasing concentration of AlkB bound to AlkG for the catalytic conversion of *n*-octane to 1-octanol. The red curve is resulted from the fitting using an apparent two-site binding model.

**Figure 7:**
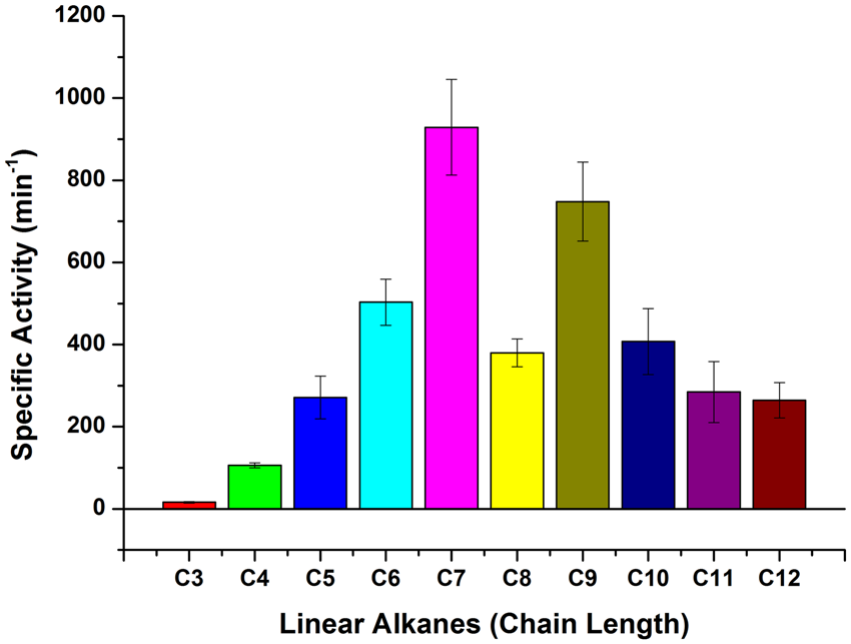
Specific activity in turnover frequency (TOF) for the electrochemical conversion of medium-chain length *n*-alkanes (C_3_–C_12_) to primary alcohols mediated by AlkB-enriched membrane bound to AlkG immobilized on a SPCE.

The effective surface coverage ratio of AlkG for DET from a SPCE to AlkG is within 0.5–2%^48^. Data obtained from catalytic conversion mediated by SPCE electrodes show that the apparent TOFs (based on the amount of AlkG placed on top of SPCE instead of electroactive AlkG, i.e. the effective surface coverage ratio of AlkG) of C_5_–C_12_ range within 250–1000 min^−1^ per unit AlkG, where the concentration ratio of AlkB to AlkG is about 2 (Fig. 6; confer Figures S6 and S7 in Supplementary Information for GC chromatograms). The specific activities (in TOF) for the conversion of C_6_, C_7_ and C_9_ *n*-alkanes to primary alcohols are in the range of 500–1,000 min^−1^ (Fig. 7). The TOFs for the conversion of C_5_, C_8_ and C_10_−C_12_ *n*-alkanes to corresponding primary alcohols are within 250–400 min^−1^. Herein, unprecedented results for the conversion of gaseous propane (C_3_) and *n*-butane (C_4_) to 1-propanol and 1-butanol, respectively, are noted; TOFs for the gas-to-liquid (GTL) conversion are 16 ± 2 and 106 ± 6 min^−1^, respectively.

## Discussion

The analysis of XANES spectra obtained from AlkG_oxidized_, AlkG_reduced_, AlkB_oxidized_ and AlkB–AlkG pair yields the plausible electron transfer pathway employed by AlkB through the transition of AlkB_oxidized_AlkG_oxidized_ → AlkB_oxidized_AlkG_reduced_ → AlkB_reduced_AlkG_reduced_, further facilitating the hydroxylation of *n*-alkanes (Fig. 1). This revelation allows the development of a system for artificial *n*-alkane hydroxylation using AlkG immobilized SPCE, in which AlkT is replaced by the electrode. Therefore, the reaction can be controlled via the supply of electrons; both AlkT and the costly reducing equivalent are no longer required.

The kinetics data suggest that one AlkG with two Fe–4 S electron transfer centres can bind with two membrane-bound AlkB to form an AlkG:2AlkB adduct, facilitating an efficient conversion of *n*-octane to 1-octanol. With a high concentration of AlkB-enriched membrane binding to AlkG, conformational changes or molecular assembly (presumably, dimerization), might not greatly vary the dissociation constant of AlkB–AlkG adduct. However, the catalytic efficiency driven by AlkB–AlkG adduct with excess of AlkB significantly improves, i.e. more than two-fold. This result agrees with the observation that the ratio for the expressed AlkB to AlkG in *P*. *putida* GPo1 and *E*. *coli* under the regulation of *alk* operon are within the range of 1:0.34 and 1:0.08–0.10, respectively^16^. The alkane hydroxylation activity given by *P*. *putida* GPo1 is usually higher than that obtained from the *alkBGT* expression in *E*. *coli*.

It is noted that differences in the specific activity among all chain length linear alkanes tested are varied; there is no particular relationship between the specific activity and the chain length of alkanes. This phenomenon may be attributed to that the recombinant AlkB is heterogeneously expressed on the membrane of *E*. *coli* and may cause the promiscuity in the selection of substrates^16^. The adsorption of the protein complex on the SPCE with high surface area and its intrinsic properties may also alter the shape or the morphology of the hydrophobic pocket in AlkB so that the relationship between the reaction activity and the chain length is difference from that observed from the wild type host cells.

It has been shown that *P*. *putida* GPo1 can metabolize propane and *n*-butane^51^. In addition, several mutations implemented in recombinant AlkB *in vivo* demonstrate the feasibility in the conversion of *n*-butane to 1-butanol^52^. In this study, the electrocatalytic conversion of gaseous propane and *n*-butane to 1-propanol and 1-butanol, respectively, is shown. To date, there is no direct chemical method reported for the efficient oxidation of C_3_−C_4_ *n*-alkanes to the corresponding primary alcohols. The catalytic process presented is environmentally benign and, in principle, can combine with the solar energy/photovoltaic cell for the sustainable conversion of either natural gas or LPG to the corresponding primary alcohols. Presumably, other small oxygenated products, such as propylene oxide or butene oxide, that are classified as fundamentally basic chemicals for the future supply in chemical industry can be generated through a similar approach^53, 54^.

In this study, spectroscopic and electrochemical evidence is presented to show that an iron-sulfur protein, AlkG, can efficiently transfer electrons towards the non-heme iron monooxygenase, AlkB, for the subsequent conversion of medium-chain length *n*-alkanes to primary alcohols. Immobilized AlkG on SPCE can interact with the AlkB-enriched membrane to form a complex for efficient conversion of C_5_−C_12_ *n*-alkanes to primary alcohols with the specific activity in TOF of 250–1000 min^−1^. Structural distortion of protein complex upon the adsorption on the surface of electrode might cause the promiscuity towards substrates, leading to the interaction with propane and *n*-butane. The proof of concept in this study provides the future feasibility in the production of green alcohols that can potentially serve as feedstock for chemical industry.

## Methods

### Materials

All chemicals were purchased from Sigma-Aldrich and were used as received unless otherwise specified. The gas cylinders of propane and *n*-butane were obtained from Huei Chyi Gas Co., Ltd. The vectors pET-21a(+) and pACYCDuet-1 were purchased from Novagen. *Escherichia coli* strain DH5α used as a carrier for the plasmid constructs and strain BL21 used for expression of plasmid constructs were purchased from Bioman Scientific Co. Ltd. (Taiwan). Primers were synthesized by Tri-I Biotech Inc. (Taiwan). UV-visible spectra were recorded on a HP 8453 diode array spectrometer and the path length for the UV-vis cuvette is 1 cm. All electrochemical experiments were carried out using a CH Instrument electrochemical workstation (CHI 611 C). The three-electrode system contains a SPCE (5 mm in diameter and 0.196 cm^2^ in area; Zensor R&D, Taiwan), an Ag/AgCl reference electrode and a platinum wire counter electrode. The working electrode was anodized prior to use by applying a potential at 2.0 V versus Ag*/*AgCl. The iron quantification was conducted by Inductively Coupled Plasma Optical Emission Spectrometer (ICP-OES 720S) (Agilent Technology). In the series of C_5_−C_12_ alkane/alcohol analysis, gas chromatography (GC, Agilent HP6890 plus) was equipped with a flame ionization detector (FID) and compounds administered were separated by a HP-5 capillary column (60 m × 0.25 mm × 0.25 μm film thickness). The analysis conditions were presented as the following unless stated otherwise: carrier gas, nitrogen, was at 1.0 mL/min; for the activity assay using octane as the substrate, the oven temperature was set isothermally at 120 °C for 10 min and then raised to 150 °C with the rate of 5 °C/min and hold for another 4 min; the injection port was at 300 °C in splitless mode. In the series of C_3_ and C_4_ alkane/alcohol analysis, gas chromatography (GC-2010 Plus, Shimadzu) was equipped with a barrier discharge ionization detector (BID) and compounds administered were separated by a DB-624 capillary column (30 m × 0.53 mm × 3 μm film thickness). The analysis conditions were presented as the following unless stated otherwise: carrier gas, helium, was at 1.0 mL/min; the oven temperature was set isothermally at 30 °C for 10 min and then raised to 200 °C with the rate of 10 °C/min and hold for another 5 min; the injection port was at 280 °C in split mode.

### Construction of AlkB and AlkG protein

The genomic DNA of *P*. *putida* GPo1 (ATCC 29347) was extracted using Gene-Spin Genomic DNA kit (Protech). The PCR amplification of the construct *alkB* was achieved by using Deep VentR DNA polymerase (New England Biolabs) and the following four primers: 5′-AAT TCA TGC TTG AGA AAC ACA GA-3′; 5′-CAT GCT TGA GAA ACA CAG AGT TCT-3′; 5′-TCG AGA TAC GAT GCT ACC GCA-3′ and 5′-GAT ACG ATG CTA CCG CAG-3′. The cohesive-end *alkB* gene with EcoR I and Xho I site was generated, cloned into the corresponding restriction sites of pET-21a( + ) and then sequenced. The Strep II tag (WSHPQFEK) and a linker (SA) were added to the *C*-terminus by a two-step PCR using constructed plasmid *pET21alkB* as a template with first pair of primers: 5′-GGA ATT CCA TAT GCT TGA GAA ACA CAG AGT TCT GG-3′ and 5′-TCA TTT TTC GAA CTG CGG GTG GCT CCA AGC GCT CGA TGC TAC CGC AGA GGT ACT-3′. The resulting product was used as template for the second pair of primers: 5′-CCG CTC GAG TCA TTT TTC GAA CTG CGG GT-3′ and 5′-GGA ATT CCA TAT GCT TGA GAA ACA CAG AGT TCT GG-3′. After the digestion with restriction enzymes Nde I and Xho I, the end product was cloned into the corresponding sites of pET-21a(+) to produce the gene construct of *pET21alkBStrep* and then sequenced to confirm its integrity^55^.

The rubredoxin-2 gene, *alkG*, was amplified from the genomic DNA using the following four primers: 5′-CAT GGC TAG CTA TAA ATG CCC G-3′; 5′-GCT AGC TAT AAA TGC CCG GA-3′; 5′-AAT TCT CAC TTT TCC TCG TAG AGC-3′ and 5′-CTC ACT TTT CCT CGT AGA GCA C-3′. The cohesive-end *alkG* gene with Nco I and EcoR I sites was generated, cloned into the corresponding restriction sites of pACYCDuet-1 and the *pACYCDuetalkG* construct was then sequenced.

The plasmid of *pET21alkBStrep* or *pACYCDuetalkG* was transformed into *E*. *coli* BL21 (DE3). The *E*. *coli* transformant of *pET21alkBStrep* was grown in Luria-Bertani (LB) medium containing 100 mg/L of ampicillin at 37 °C in a 10 L fermentor. When the turbidity of cell culture monitored by OD_600_ reached 0.7, 60 mg/L of FeSO_4_ was supplemented and 0.4 mM of isopropyl ß-D-thiogalactopyranoside (IPTG) was added to induce the overexpression of AlkB-Strep. The temperature was then lower down to 20 °C and cell cultures were incubated for another 22 hr. The *E*. *coli* transformant of *pACYCDuetalkG* was grown in LB medium containing 34 mg/L of chloramphenicol at 37 °C in a 10 L fermentor. When OD_600_ reached 0.7, 60 mg/L of FeSO_4_ was supplemented and 0.4 mM of IPTG was added to induce the overexpression of AlkG. The temperature was then lower down to 20 °C and cell cultures were incubated for another 22 hr.

### Purification of AlkB protein

All procedures for the isolation of AlkB-enriched membrane and the purification of AlkB were performed at 4 °C. Cells were resuspended in 50 mM Tris-HCl containing 10 μg/mL of DNaseI, pH 7.5, and disrupted in a pre-chilled French pressure cell (SLM Aminco) via three passages at 20,000 lb/in ref. 2. The lysate was subjected to centrifugation at 12,000 rpm for 20 min to remove cell debris. Subsequently, the supernatant was subjected to ultracentrifugation at 36,000 rpm for 1 hr and the AlkB-enriched membrane portion was collected after the ultracentrifugation.

To purify Strep-tagged AlkB, the collected membrane portion together with the pellet portion was solubilized in 50 mM Tris-HCl, 300 mM NaCl, 1 mM FeSO_4_, 0.23% *N*,*N*-dimethyldodecylamine *N*-oxide (LDAO, Sigma), pH 7.5, homogenized and then stirred in a beaker for 1 hr. Followed by the solubilization, the membrane extract was diluted with 50 mM Tris-HCl, 300 mM NaCl, 1 mM FeSO_4_, pH 7.5, to reduce the concentration of LDAO to 0.07%. The membrane extract was subjected to centrifugation at 12,000 rpm for 20 min and the supernatant was loaded onto a Strep-Tactin column (GE Healthcare) running by gravity flow. The unbound was washed with five bed volumes of 50 mM Tris-HCl, 300 mM NaCl, 0.07% LDAO, pH 7.5, and proteins were eluted with two bed volumes of the same buffer containing 0.5 mM D-desthiobiotin (Figure S8 in Supplemental Information). The AlkB-containing fractions were pooled and concentrated using a concentrator (Vivaspin 20, MW cutoff 30 kDa, GE Healthcare). Protein concentrations were determined through Bio-Rad DC protein assay (Bio-Rad) using bovine serum albumin as the standard. The purified AlkB can be stored at 4 °C for a day and the activity diminishes afterward.

### Purification of AlkG protein

All procedures were performed at 4 °C. Cells were resuspended in 50 mM Tris-HCl containing 1 mM of phenylmethylsulfonyl fluoride (PMSF) and 10 μg/mL of DNaseI, pH 7.5, and then disrupted in a pre-chilled French pressure cell (SLM Aminco) via three passages at 20,000 lb/in2. The lysate was subjected to ultracentrifugation at 36,000 rpm for 1 hr. Ammonium sulfate was slowly added to the supernatant until the final concentration reaches 20% and then centrifuged at 6,000 rpm for 15 min to remove the contaminating proteins in the precipitate. The clear reddish solution was concentrated and dialyzed against 1 L of 50 mM Tris-HCl, pH 7.5, for three times to remove the excess salt. The resulting solution was loaded onto a HiLoad Q Sepharose Fast Flow column (GE Healthcare) equilibrated with 50 mM Tris-HCl, pH 7.5. An ascending linear gradient of sodium chloride (0–1 M) was applied and AlkG-containing fractions were pooled. Ammonium sulfate was added into the pooled solution until the final concentration reaches 35% and then centrifuged at 6,000 rpm for 15 min to remove the contaminating proteins in the precipitate. The clear reddish solution part was loaded onto a Phenyl Sepharose HP column (GE Healthcare) equilibrated with 50 mM Tris-HCl, 35% ammonium sulfate, pH 7.5. A descending linear gradient of ammonium sulfate (35–0%) was applied and eluted fractions containing active AlkG were pooled. The purified AlkG was dialyzed overnight against 50 mM Tris-HCl, pH 7.5 and then concentrated. The concentration of AlkG was determined using the characteristic absorbance at 378 and 495 nm with the extinction coefficient of 12,400 and 10,600 M^−1^cm^−1^, respectively.

### Western blot analysis

Detection of Strep-tagged AlkB was achieved by Western blot analysis using Strep-Tactin HRP conjugate against the Strep tag. Proteins from membrane extracts were separated by SDS-PAGE and then transferred onto a hydrophobic PVDF membrane (0.45 μm Hybond-P, GE Healthcare). Strep-tagged AlkB was detected using Strep-Tactin HRP conjugate (IBA GmbH), which recognizes the *C*-terminal Strep tag attached on AlkB, following the manufacturer’s instruction. The resulting bands were visualized with the luminescence elicited from ECLTM Western blotting reagent (Bionovas Inc.) and analysed using a CCD camera system (UVP Inc.). The quantitation of the recombinant AlkB was assessed using the calibration curve established by Strep-tagged marker proteins (Bionovas Inc.)^56, 57^.

### Determination of the iron content in AlkG and Strep-tagged AlkB by ICP-OES

Aliquots (0.2 mL) of protein samples were dissolved in 4.8 mL of saturated 65% HNO_3_ solution (suprapure grade, Merck). The samples were digested in a MARS5 microwave digestion system (CEM Inc.). The temperature was stepped up incrementally from room temperature to 180 °C in 15 min, and maintained at 180 °C for another 15 min. The process of nitrate digestion was then terminated as the temperature was gradually lowered down to room temperature.

The digested samples were then diluted with 20 mL doubly distilled water (Millipore) prior to the analysis. The iron concentration of samples was determined by interpolating a linear plot of a series standard solutions of Fe(NO_3_)_2_ in 0.10 N HNO_3_. A solution of 0.10N HNO_3_ in distilled water was used as the iron-free control. The digested samples were measured by ICP-OES 720S (Agilent Technology).

### Fabrication of purified AlkG modified electrode

The purified recombinant AlkG was immobilized on the surface of a bare SPCE. The SPCE with a working area of 0.196 cm^2^ and a conductive track radius of 2.5 mm was purchased from Zensor R&D (Taichung, Taiwan). The measured average resistance is 85.64 ± 2.10 Ω/cm. The redox potential of AlkB-enriched membrane and purified AlkB solution were determined. The AlkB-enriched membrane is easier to be employed and exhibits more efficient capability in the electron transfer to oxidize a series of C_5_−C_12_ *n*-alkanes. The SPCE was pre-treated by gently washed with deionized water and then air-dried prior to the modification by protein.

Purified AlkG modified electrode was prepared by drop-coated 10 μL of 4.436 mM purified AlkG protein on a bare SPCE and air-dried for 3 hr. Then, 10 μL of 0.5% Nafion^®^/MeOH solution was coated onto as-prepared AlkG protein modified electrode and the modified electrode was subsequently stored at 4 °C for 1 hr.

### Electrochemical hydroxylation of *n*-alkanes, determination of apparent turnover number (TON) and turnover frequency (TOF)

Electrochemical measurements were performed with a CHI 611C electrochemical workstation in a three-electrode cell assembly. A Nafion®/AlkG modified SPCE working electrode, an Ag/AgCl, 3 M KCl reference electrode and a platinum auxiliary electrode were used to complete the cell setup. The Nafion®/AlkG modified SPCE was employed in the electrochemical hydroxylation of C_3_–C_12_ *n*-alkanes (C_5_−C_12_: 0.4–1.1 mM; C_3_ and C_4_: 1.0 mL condensed liquid by dry ice) under a potential of −0.6 V versus Ag/AgCl in the presence of 427 μM AlkB-enriched membrane in the electrochemical cell.

In the series of C_5_−C_12_ alkane/alcohol analysis, after the conversion, the reaction mixtures were transferred to a 1.5-mL Eppendorf tube and extracted with 0.005% *p*-xylene in 100 μL methylene solution, in which *p*-xylene was served as an internal standard. Aliquots of 0.050, 0.10, 0.21, 0.41 and 0.82 mg of primary C_5_−C_12_ alcohols were dissolved, respectively, in 100 μL of *p*-xylene containing methylene chloride. The corresponding GC intensities of primary alcohols were normalized by the intensity derived from the internal standard, *p*-xylene. In the series of C_3_ and C_4_ alkane/alcohol analysis, methyl *t*-butyl ether (MTBE) was employed as an internal standard with the addition of 1 μL MTBE into 1 mL aqueous solution. The calibration curve was established by adding aliquots of 0.15, 0.29, 0.44, 0.59, 1.76, 2.93 and 5.85 mg of primary C_3_−C_4_ alcohols into ddH_2_O (1 mL) containing 0.1% MTBE.

The amount of product (primary alcohols) formation is determined through gas chromatography using the established calibration curve. The apparent turnover number (TON) is calculated by dividing the mole of product formation by the mole of AlkG, as the limiting factor is AlkG. The apparent turnover frequency (TOF) is obtained by dividing TON by the duration of electroanalysis, i.e. 30 min.

### X-Ray absorption spectroscopy

X-ray absorption data were collected at the National Synchrotron Radiation Research Center in Hsinchu, Taiwan (Beamline Wigger 17C1) using a Si(111) double crystal monochromator in the region of the Fe K-edge (the energy calibration is set with Fe foil at﻿﻿ 7112 eV). Protein samples were loaded onto either a sample holder (1.4 cm × 1.4 cm × 0.2 cm) covered with sheets of kapton or sealed in a polyethylene bag. During measurements, the samples were kept at 277 K (cooling by cold air device). The data collection was carried out in combination with the sleeping mode (i.e., without shining x-ray on the sample) for 4–18 s after every 6–24 s of X-ray irradiation. Samples tested were sufficiently thin to allow the total penetration of incident X-ray beam. Fluorescence data were collected on an argon-filled ionization chamber. Under these conditions, the edge jump could be regarded as a measure for the content of the corresponding absorbing element in the sample.

The background subtraction and normalization of XAS data were implemented in the program ATHENA. The pre-edge peak areas or intensities were calculated followed the method established by Roe, A. L. *et al*.^41^, and the peak was isolated from the normalized XAS spectra after the subtraction of a sigmoidal function from a nonlinear curve fit of the raw data using the program OriginPro 8.6.0 (OriginLab Co.). After background subtraction, the area of pre-edge peak was obtained from the peak integration at 7108–7118 eV (AlkG_oxidized_ and AlkG_reduced_) or 7110–7120 eV (AlkB_oxidized_). The pre-edge peak of AlkB_oxidized_ at ca. 7114.5 eV was further deconvoluted into two Gaussian functions^36^ using “Multiple Peak Fit” and “Simulate Curve” of OriginPro 8.6.0. It is noteworthy that many previous characterization studies using XANES in iron–sulfur and non-heme iron proteins including SdhC, PDO, MMOH, TMOH do not usually provide the detailed data to specify the mid-point K-edge and pre-edge energy. To extract the corresponding information from the original XAS spectra in the reported literatures^34, 35, 37, 38^, the converted image files from the pdf documents were digitalized and reproduced by a function of “Digitize Image” in OriginPro 8.6.0. The obtained raw data were further processed and normalized to obtain the K-energy at the midpoint of absorption edge as well as the pre-edge peaks were identified by the same program. For all samples presented in this study, no photoreduction or photodamage was observed from the comparison between the first and the last spectra collected from a given sample. The fitting of experimental extended X-ray absorption fine structure (EXAFS) data was done by a nonlinear least-square fitting algorithm implemented by the IFEFFIT program^58–60^. Data fitting quality was evaluated with the goodness-of-fit factor defined as:

2 $${R}_{fit}=\frac{{\sum }_{i=1}^{n}\,\{{[Re({f}_{i})]}^{2}+{[Im({f}_{i})]}^{2}\}}{{\sum }_{i=1}^{n}\,\{{[Re({\tilde{\chi }}_{datai})]}^{2}+{[Im({\tilde{\chi }}_{datai})]}^{2}\}},$$

where *χ* = *k* ^3^ *χ* and *n* is the number of evaluations of *f*_*i*_, with ${f}_{i}={\tilde{\chi }}_{{\rm{data}}i}\mbox{--}{\tilde{\chi }}_{{\rm{model}}i}$ (and hence *R*_fit_) minimized in the nonlinear least-square fitting algorithm.

## Electronic supplementary material

Supporting Information

## References

[1] Thomas JM, Raja R, Sankar G, Bell RG (1999). Molecular-sieve catalysts for the selective oxidation of linear alkanes by molecular oxygen. Nature.

[2] Munz D, Strassner T (2015). Alkane C-H functionalization and oxidation with molecular oxygen. Inorg. Chem..

[3] Shul’pin, G. B. New trends in oxidative functionalization of carbon-hydrogen bonds: a review. *Catalysts* **6** (2016).

[4] Van de Vyver S, Roman-Leshkov Y (2013). Emerging catalytic processes for the production of adipic acid. Catal. Sci. Technol..

[5] Sun M (2014). Catalytic Oxidation of Light Alkanes (C_1_–C_4_) by Heteropoly Compounds. Chem. Rev..

[6] Austin RN, Groves JT (2011). Alkane-oxidizing metalloenzymes in the carbon cycle. Metallomics.

[7] Rojo F (2009). Degradation of alkanes by bacteria. Environ. Microbiol..

[8] Ji YR, Mao GN, Wang YY, Bartlam M (2013). Structural insights into diversity and *n*-alkane biodegradation mechanisms of alkane hydroxylases. Front. Microbiol..

[9] van Beilen JB, Funhoff EG (2007). Alkane hydroxylases involved in microbial alkane degradation. Appl. Microbiol. Biotechnol..

[10] Shanklin J, Cahoon EB (1998). Desaturation and related modifications of fatty acids. Annu. Rev. Plant Physiol. Plant Mol. Biol..

[11] Baptist JN, Gholson RK, Coon MJ (1963). Hydrocarbon oxidation by a bacterial enzyme system. I. Products of octane oxidation. Biochim. Biophys. Acta.

[12] Peterson JA, Basu D, Coon MJ (1966). Enzymatic omega-oxidation.I. Electron carriers in fatty acid and hydrocarbon hydroxylation. J. Biol. Chem..

[13] Peterson JA, Kusunose M, Kusunose E, Coon MJ (1967). Enzymatic omega-oxidation. II. Function of rubredoxin as the electron carrier in omega-hydroxylation. J. Biol. Chem..

[14] Ruettinger RT, Olson ST, Boyer RF, Coon MJ (1974). Identification of the omega-hydroxylase of *Pseudomonas oleovorans* as a nonheme iron protein requiring phospholipid for catalytic activity. Biochem. Biophys. Res. Commun.

[15] Ueda T, Lode ET, Coon MJ (1972). Enzymatic omega-oxidation. 6. Isolation of homogeneous reduced diphosphopyridine nucleotide-rubredoxin reductase. J. Biol. Chem..

[16] Staijen IE, van Beilen JB, Witholt B (2000). Expression, stability and performance of the three-component alkane mono-oxygenase of *Pseudomonas oleovorans* in *Escherichia coli*. Eur. J. Biochem..

[17] Lode ET, Coon MJ (1971). Enzymatic omega-oxidation. V. Forms of *Pseudomonas oleovorans* rubredoxin containing one or two iron atoms: structure and function in omega-hydroxylation. J. Biol. Chem..

[18] Shanklin J, Achim C, Schmidt H, Fox BG, Munck E (1997). Mössbauer studies of alkane omega-hydroxylase: Evidence for a diiron cluster in an integral-membrane enzyme. Proc. Natl. Acad. Sci. USA.

[19] McKenna EJ, Coon MJ (1970). Enzymatic omega-oxidation. IV. Purification and properties of the omega-hydroxylase of *Pseudomonas oleovorans*. J. Biol. Chem..

[20] Austin, R. N., Born, D., Lawton, T. J. & Hamilton, G. E. In *Hydrocarbon and Lipid Microbiology Protocols* 1-15 (Humana Press, 2015).

[21] Alonso H, Roujeinikova A (2012). Characterization and two-dimensional crystallization of membrane component AlkB of the medium-chain alkane hydroxylase system from *Pseudomonas putida* GPo1. Appl. Environ. Microbiol..

[22] Alonso H (2014). Structural and mechanistic insight into alkane hydroxylation by *Pseudomonas putida* AlkB. Biochem. J..

[23] Cooper HLR (2012). Parallel and competitive pathways for substrate desaturation, hydroxylation, and radical rearrangement by the non-heme diiron hydroxylase AlkB. J. Am. Chem. Soc..

[24] Fu H, Newcomb M, Wong CH (1991). *Pseudomonas olevorans* monooxygenase catalyzed assymetric epoxidation of allyl alcohol derivatives and hydroxylation of a hypersensitive radical probe with the radical ring-opening rate exceeding the oxygen rebound rate. J. Am. Chem. Soc..

[25] Austin RN (2008). Cage escape competes with geminate recombination during alkane hydroxylation by the diiron oxygenase AlkB. Angew. Chem. Int. Ed..

[26] Austin RN, Chang HK, Zylstra GJ, Groves JT (2000). The non-heme diiron alkane monooxygenase of *Pseudomonas oleovorans* (AlkB) hydroxylates via a substrate radical intermediate. J. Am. Chem. Soc..

[27] Ping JF, Wang YX, Ying YB, Wu J (2012). Application of Electrochemically Reduced Graphene Oxide on Screen-Printed Ion-Selective Electrode. Anal. Chem..

[28] Kong FY (2014). A paper disk equipped with graphene/polyaniline/Au nanoparticles/glucose oxidase biocomposite modified screen-printed electrode: Toward whole blood glucose determination. Biosens. Bioelectronics.

[29] Molinero-Abad B, Alonso-Lomillo MA, Dominguez-Renedo O, Arcos-Martinez MJ (2014). Sulfite oxidase biosensors based on tetrathiafulvalene modified screen-printed carbon electrodes for sulfite determination in wine. Anal. Chim. Acta..

[30] Wang J (2008). Electrochemical glucose biosensors. Chem. Rev..

[31] Willner I, Katz E (2000). Integration of layered redox proteins and conductive supports for bioelectronic applications. Angew. Chem. Int. Ed..

[32] Perry A, Lian LY, Scrutton NS (2001). Two-iron rubredoxin of *Pseudomonas oleovorans*: production, stability and characterization of the individual iron-binding domains by optical, CD and NMR spectroscopies. Biochem. J..

[33] Perry A, Tambyrajah W, Grossmann JG, Lian LY, Scrutton NS (2004). Solution structure of the two-iron rubredoxin of Pseudomonas oleovorans determined by NMR spectroscopy and solution X-ray scattering and interactions with rubredoxin reductase. Biochemistry.

[34] Li Z (2003). X-ray Absorption Spectroscopic Analysis of Reductive [2Fe-2S] Cluster Degradation in Hyperthermophilic Archaeal Succinate:Caldariellaquinone Oxidoreductase Subunits. Biochemistry.

[35] Tsang HT, Batie CJ, Ballou DP, Penner-Hahn JE (1989). X-ray absorption spectroscopy of the [2-iron-2-sulfur] Rieske cluster in *Pseudomonas cepacia* phthalate dioxygenase. Determination of core dimensions and iron ligation. Biochemistry.

[36] Lo FC (2012). The metal core structures in the recombinant *Escherichia coli* transcriptional factor SoxR. Chem.-Eur. J..

[37] Rudd DJ (2004). Determination by X-ray absorption spectroscopy of the Fe-Fe separation in the oxidized form of the hydroxylase of methane monooxygenase alone and in the presence of MMOD. Inorg. Chem..

[38] Rudd DJ, Sazinsky MH, Lippard SJ, Hedman B, Hodgson KO (2005). X-ray absorption spectroscopic study of the reduced hydroxylases of methane monooxygenase and toluene/o-xylene monooxygenase: differences in active site structure and effects of the coupling proteins MMOB and ToMOD. Inorg. Chem..

[39] Randall CR (1995). X-ray Absorption Pre-Edge Studies of High-spin Iron(II) Complexes. Inorg. Chem..

[40] Westre TE (1997). A Multiplet Analysis of Fe K-Edge 1s → 3d Pre-Edge Features of Iron Complexes. J. Am. Chem. Soc..

[41] Roe AL (1984). X-ray Absorption Spectroscopy of Iron-Tyrosinate Proteins. J. Am. Chem. Soc..

[42] Shulman RG, Eisenberger P, Blumberg WE, Stombaugh NA (1975). Determination of the iron-sulfur distances in rubredoxin by x-ray absorption spectroscopy. Proc. Natl. Acad. Sci. USA.

[43] Shu L (1998). EXAFS and Mössbauer characterization of the Diiron(III) site in stearoyl-acyl carrier protein Δ9– desaturase. J. Biol. Inorg. Chem..

[44] True AE, Scarrow RC, Randall CR, Holz RC, Que LJ (1993). EXAFS studies of uteroferrin and its anion complexes. J. Am. Chem. Soc..

[45] Bai YH (2015). X-ray structure of a mammalian stearoyl-CoA desaturase. Nature.

[46] Scarrow RC (1987). EXAFS studies of binuclear iron proteins: hemerythrin and ribonucleotide reductase. J. Am. Chem. Soc..

[47] Shu L, Liu Y, Lipscomb JD, Que LJ (1996). X-ray absorption spectroscopic studies of the methane monooxygenase hydroxylase component from *Methylosinus trichosporium* OB3b. J. Biol. Inorg. Chem..

[48] Prodromidis MI, Florou AB, Tzouwara-Karayanni SM, Karayannis MI (2000). The Importance of Surface Coverage in the Electrochemical Study of Chemically Modified Electrodes. Electroanalysis.

[49] Wang SF (2005). Direct Electrochemistry and Electrocatalysis of Heme Proteins Entrapped in Agarose Hydrogel Films in Room-Temperature Ionic Liquids. Langmuir.

[50] Bard, A. J. & Faulkner, L. R. *Electrochemical Methods*, *Fundamentals*, *and Applications*. 2nd ed., (Wiley, 2001).

[51] Johnson EL, Hyman MR (2006). Propane and *n*-butane oxidation by *Pseudomonas putida* GPo1. Appl. Environ. Microbiol..

[52] Koch DJ, Chen MM, Van Beilen JB, Arnold FH (2009). *In vivo* evolution of butane oxidation by terminal alkane hydroxylases AlkB and CYP153A6. Appl. Environ. Microbiol..

[53] Peters M (2011). Chemical Technologies for Exploiting and Recycling Carbon Dioxide into the Value Chain. ChemSusChem.

[54] Marimuthu A, Zhang J, Linic S (2013). Tuning selectivity in propylene epoxidation by plasmon mediated photo-switching of Cu oxidation state. Science.

[55] Ramu R (2011). Regio-selective hydroxylation of *gem*-difluorinated octanes by alkane hydroxylase (AlkB). Tetrahedron Lett..

[56] Bernaudat F (2011). Heterologous expression of membrane proteins: choosing the appropriate host. PLoS One.

[57] Walker, J. M. *The Protein Protocols Handbook*. 2nd ed., 421–428 (Humana Press, 2002).

[58] Ravel B, Newville M (2005). ATHENA, ARTEMIS, HEPHAESTUS: data analysis for X-ray absorption spectroscopy using IFEFFIT. J. Synchrotron Rad..

[59] Newville M (2001). IFEFFIT: interactive XAFS analysis and FEFF fitting. J. Synchrotron Rad..

[60] Ravel B (2001). ATOMS: crystallography for the X-ray absorption spectroscopist. J. Synchrotron Rad..

