# Supplementary material for: Electrochemical Hydroxylation of C3–C12n-Alkanes by Recombinant Alkane Hydroxylase (AlkB) and Rubredoxin-2 (AlkG) from Pseudomonas putida GPo1
Source: Sci Rep. 2017 Aug 21;7:8369. doi: 10.1038/s41598-017-08610-w (PMC5566439; doi:10.1038/s41598-017-08610-w)
Supplement: Supplementary file 1 — Supporting Information [file 41598_2017_8610_MOESM1_ESM.pdf]

# Supplementary Information

Electrochemical Hydroxylation of $C_3$–$C_{12}$ *n*-Alkanes by Recombinant Alkane Hydroxylase (AlkB) and Rubredoxin-2 (AlkG) from *Pseudomonas putida* GPo1

Yi-Fang Tsai[+,1], Wen-I Luo[+,1], Jen-Lin Chang[2], Chun-Wei Chang[1], Huai-Chun Chuang[1], Ravirala Ramu[1], Guor-Tzo Wei[3], Jyh-Myng Zen[*,2], and Steve S.-F. Yu[*,1]

[1]Institute of Chemistry, Academia Sinica, Taipei 115, Taiwan
[2]Department of Chemistry, National Chung Hsing University, Taichung 402, Taiwan
[3]Department of Chemistry and Biochemistry, National Chung Cheng University, Chia-yi 621, Taiwan
+These authors contribute equally to this work
*Correspondence should be addressed to Steve S.-F. Yu (sfyu@gate.sinica.edu.tw) and Jyh-Myng Zen (jmzen@dragon.nchu.edu.tw)

## Supplementary Information

Soluble non-heme diiron enzymes can be categorized into four classes (Curr. Opin. Struct. Biol., 1995, 5, 578) and they are related both in the aspect of sequence and structure (ChemBioChem, 2001, 2, 583):

| Class | | Protein |
|---|---|---|
| Helix-bundle proteins | I. Large helix-bundle proteins | Ribonucleotide reductase R2, methane monooxygenase, stearoyl-acyl protein $\Delta^9$-desatuarse, toluene hydroxylase, phenol hydroxylase |
| | II. Simple helix-bundle proteins with overhead connection | Ferritin, bacterioferritin, rubrerythrin |
| | III. Simple helix-bundle proteins without overhead connection | Hemerythrin, myohemerythrin |
| $\alpha/\beta$ sandwich structures | | Purple acid phosphatase |

Primary sequence-wise, soluble non-heme diiron proteins can be further divided into two groups: Class I and Class II (Biochemistry, 1994, 33, 12776). Class I non-heme diiron proteins, such as hemerythrin (Hr) and purple acid phosphatase, contain $HX_3E$, $HX_3H$, and $HX_4D$ motif. Class II non-heme diiron proteins contains diiron proteins such as soluble fatty acid desaturase, soluble methane monooxygenase (MMOH), ribonucleotide reductase (RNR-R2), rubrerythrin and toluene-4-monooxygenase (ToMOH) (Annu. Rev. Plant. Physiol. Plant. Mol. Biol., 1998, 49, 611). Primary sequence alignment shows that soluble fatty acid desaturase, MMOH and RNR-R2 share a consensus $[D(E)EX_2H]_2$ motif for the coordination of the diiron active site.

However, the consensus $[D(E)EX_2H]_2$ motif found in Class II soluble diiron proteins does not exist in integral membrane bound non-heme diiron proteins such as integral membrane fatty acid desaturase (for instance, integral membrane stearoyl-acyl carrier protein $\Delta^9$ desaturase, SCD1), xylene monooxygenase (XylM) and AlkB (Annu. Rev. Plant. Physiol. Plant. Mol. Biol., 1998, 49, 611). Instead, nine conserved histidine residues are found. This class of non-heme diiron proteins is distinct from the soluble non-heme diiron proteins and the diiron active site is ligated by multiple histidine residues.

Primary sequence alignment of AlkB, XylM and SCD1 is shown below. The green coded partial conserved aspartic acid, D181, on AlkB is shown to be related to enzyme activity as the mutation at this residue leads to the loss of activity (FEBS Lett., 2003, 545, 188). The magenta coded E143, D165 and E229 on SCD1 are known to be involved in the secondary coordination sphere of diiron active site (Nature, 2015,524, 252). The blue coded conserved histidine (H273) on AlkB is essential for alkane oxidation (J. Bacteriol., 2005, 187, 85); all other conserved histidine residues are coded in yellow.

```
AlkB    -----------mlekhrvldsapeyvdkkkylwilstlwpatpmigiwlanetgwgifyg
XylM    -------------------------mdtlryyli-----pvvtacgli-------gfyyg
SCD1    mpahmlqeisssytttttitappsgnerekvktv-----plhleedir------------

AlkB    lvllvwyga-----lplldamfgedfnnppeevvpklekeryyrvlt-yltvpmhyaali
XylM    -gywvwlgaatfpalmvldvilpkdfs--arkvsp------ffadltqylqlplmiglyg
SCD1    --------------pemkedihdptyqd-eegpppkley--vwrni--ilmvllhlggl-

AlkB    vsawwvgtqpmsw---leigalal-------------slgivngl-alntghelghkket
XylM    llvfgvengrielseplqvagcil-------------slawlsgvptlpvshelmhrrhw
SCD1    ---ygi----------ilvpscklytclfgifyymtsalgitaga------hrlwshrty

AlkB    fdrwmakivla------vvgyghffiehnkghhrdvatpmdpatsrmg---esiykfsir
XylM    lprkmaqllam------fygdpnrdiahvnthhlyldtpldsdtpyrg---qtiysfvis
SCD1    karlplrifliiantmafqndvyewardhrahhkfsethadphnsrrgfffshvgwllvr

AlkB    eipgafirawgleeqrlsrrgqsvwsfdneilqp--miitvilyavll-alfgpkmlvfl
XylM    atvgsvkdaikieaetlrrkgqspwnlsnktyqy--valllalpglvs-ylggpalglvt
SCD1    khpavkekggkldmsdl--kaeklvmfqrryykpglllmcfilptlvpwycwgetfvnsl

AlkB    piqmafgwwqltsanyieh-----ygllrqkmedgryehqkp---hhswnsnhivsnlvl
XylM    iasmiiakgivegfnyfqh-----yglvr--------dldqpillhhawnhmgtivrplg
SCD1    fvstflrytlvlnatwlvnsaahlygyrp-------ydkniq-------srenilvslga

AlkB    fhlqrhsdhhahptrsyqslrdfpglpalptgypgaf-lmamipq-wfrsvmdpkvvdw-
XylM    ceitnhinhhidgytrfyelrpekeapqmpslfv-cf-llglipplwfaliakpklrdwd
SCD1    vgegfhnyhhtfpfd--ysaseyrwhinfttffidcmaalglayd--rkkvskatvlari

AlkB    ---aggdlnkiqiddsmretylkkfgtssaghssstsavas
XylM    qryatpgerelamaankkagw-plwceselgrvasi-----
SCD1    kr-tgdgshkss-----------------------------
```

The comparison of structural data (either XRD or XAS) for non-heme diiron enzymes is listed in Table S1 and S2:

**Table S1**. Soluble non-heme diiron proteins.

| Non-heme diiron enzyme | Redox state | Approach | Fe-Fe R(Å) | Reference & Note |
|---|---|---|---|---|
| Ferritin | $Fe^{III}$-$Fe^{III}$ | X-Ray Diffraction | 3.2 | JMB, 2001, 307, 587 Note: *E.coli* |
| Bacterioferritin | $Fe^{II}$-$Fe^{II}$ | X-Ray Diffraction | 3.7 | JACS, 2009, 131, 6808 Note: *E.coli* |
| | $Fe^{III}$-$Fe^{III}$ | X-Ray Diffraction | 3.6 | |
| Hemerythrin (Hr) | $Fe^{II}$-$Fe^{II}$ deoxyHr | X-Ray Diffraction | 3.3 | Chem. Rev., 1994, 94, 715 |
| | | EXAFS | 3.6 | Biochemistry, 1988, 27, 7470 |
| | $Fe^{III}$-$Fe^{III}$ oxyHr | X-Ray Diffraction | 3.3 | Chem. Rev., 1994, 94, 715 |
| | | EXAFS | 3.2 | Chem. Rev., 1994, 94, 715 Biochemistry, 1988, 27, 7470 |
| | $Fe^{III}$-$Fe^{III}$ metHr | X-Ray Diffraction | 3.2 | Chem. Rev., 1994, 94, 715 |
| | | EXAFS | 3.1 | Chem. Rev., 1994, 94, 715 Biochemistry, 1988, 27, 7470 |
| | $Fe^{III}$-$Fe^{III}$ azidometHr | X-Ray Diffraction | 3.2 | Chem. Rev., 1994, 94, 715 |
| | | EXAFS | 3.2 | Biochemistry, 1988, 27, 7470 |
| | | EXAFS | 3.4 | PNAS, 1982, 79, 6255 |
| Methane mono-oxygenase hydroxylase (MMOH) | $Fe^{II}$-$Fe^{II}$ | X-Ray Diffraction | 3.3 | Chem. Biol., 1995, 2, 409 |
| | | EXAFS | 3.3 | Inorg. Chem., 2005, 44, 4546 |
| | $Fe^{III}$-$Fe^{III}$ | X-Ray Diffraction | 3.1 | Chem. Biol., 1995, 2, 409 |
| | | EXAFS | 3.0 | Inorg. Chem., 2004, 43, 4579 |
| | $Fe^{IV}$-$Fe^{IV}$ | EXAFS | 2.5 | Science, 1997, 275, 515 |

**Table S1.** Soluble non-heme diiron proteins (cont'd).

| Non-heme diiron enzyme | Redox state | Approach | Fe-Fe R(Å) | Reference & Note |
|---|---|---|---|---|
| Purple acid phosphatase (PAP), mammalian | $Fe^{II}$-$Fe^{III}$ | EXAFS | 3.5 | JACS, 1993, 115, 4246<br>Note: also termed tartrate-resistant acid phosphatase (TRAP) |
| | $Fe^{III}$-$Fe^{III}$ | X-Ray Diffraction | 3.5[1]<br>3.3[2] | [1]JMB, 1999, 290, 201<br>JMB, 1999, 291, 135<br>[2]Structure, 1999, 7, 757<br>JMB, 2005, 351, 233 |
| Ribonucleotide reductase, R2 subunit (RNR-R2) | $Fe^{II}$-$Fe^{II}$ | X-Ray Diffraction | 3.9 | Structure, 1996,4, 1053 |
| | $Fe^{III}$-$Fe^{III}$ | X-Ray Diffraction | 3.3 | JMB, 1993, 232, 123<br>JACS, 1998, 120, 849 |
| | | EXAFS | 3.2 | JACS, 1987,109, 7857 |
| | $Fe^{III}$-$Fe^{IV}$ | EXAFS | 2.5 | JACS, 1998, 120, 849<br>Note: intermediate X |
| Rubrerythrin, diiron site | $Fe^{II}$-$Fe^{II}$ | X-Ray Diffraction | 4.0 | JACS, 2002, 124, 9845 |
| | $Fe^{III}$-$Fe^{III}$ | | 3.3 | |
| Soluble aldehyde decarbonylase | - | X-Ray Diffraction | 3.3 | Science, 2010, 329, 559<br>ACS Chem. Biol., 2014, 9, 2584 |
| Soluble fatty acid desaturase, stearoyl-acyl carrier protein $\Delta^9$ desaturase | $Fe^{II}$-$Fe^{II}$ | X-Ray Diffraction | 4.2 | EMBO J, 1996, 15, 4081<br>JBC, 2003, 278, 25072<br>Note: castor |
| | $Fe^{III}$-$Fe^{III}$ | X-Ray Diffraction | 3.2 | JBC, 2007, 282, 19863<br>Note: ivy |
| | | EXAFS | 3.1 | JBIC, 1998, 3,392<br>Note: castor<br>Note: major species, Fe-Fe = 3.1 Å; minor species, Fe-Fe = 3.4 Å |
| Toluene mono-oxygenase (ToMOH) | $Fe^{II}$-$Fe^{II}$ | EXAFS | 3.4 | Inorg. Chem., 2005, 44, 4546 |
| | $Fe^{III}$-$Fe^{III}$ | X-Ray Diffraction | 3.0 | JBC, 2004, 279, 30600 |

**Table S2**. Integral membrane non-heme diiron proteins.

| Non-heme diiron enzyme | Redox state | Approach | Fe-Fe R(Å) | Reference & Note |
|---|---|---|---|---|
| Integral membrane fatty acid desaturase, stearoyl-acyl carrier protein $\Delta^9$ desaturase (SCD1) | - | X-Ray Diffraction | - | Nat. Struct. Mol. Biol., 2015, 22, 581 <br> Note: Zn was incorporated instead of Fe |
| | - | X-Ray Diffraction | 6.4 Zn-Zn | Nature, 2015, 524, 252 <br> Note: Zn was incorporated instead of Fe |
| Xylene mono-oxygenase (XylM) | - | - | - | Note: no structural information available |
| AlkB | $Fe^{III}$-$Fe^{III}$ | EXAFS | This work | |

(a) (b)

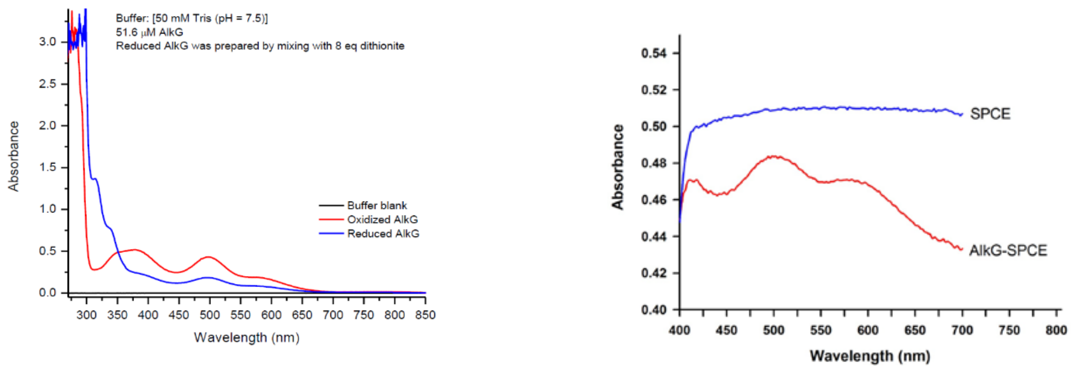


**Figure S1.** UV-vis spectra of: **(a)** Oxidized AlkG (red) and partially reduced AlkG by sodium dithionite (blue) in 50 mM Tris, pH 7.5; buffer blank is in black. **(b)** Screen-printed carbon electrode (SPCE, blue) and oxidized AlkG immobilized on SPCE (red).

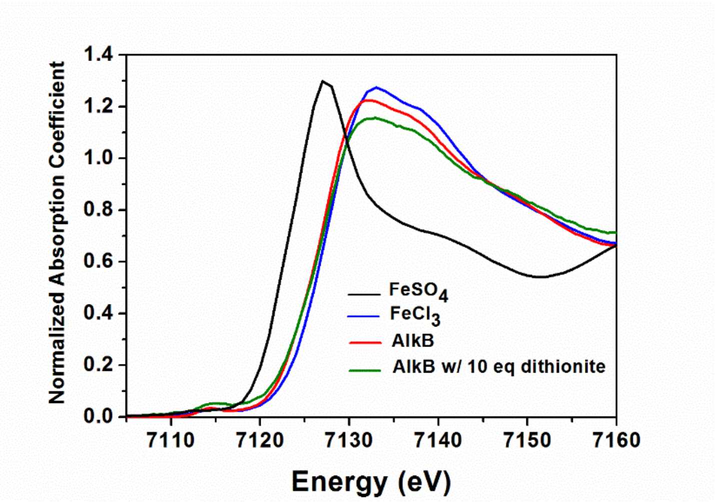


**Figure S2**. X-ray absorption near edge structure (XANES) spectra of purified AlkB. It is indicated that oxidized AlkB is obtained and the addition of dithionite only slightly alters the oxidation state of AlkB (10 eq. of dithionite is supplemented; green line).

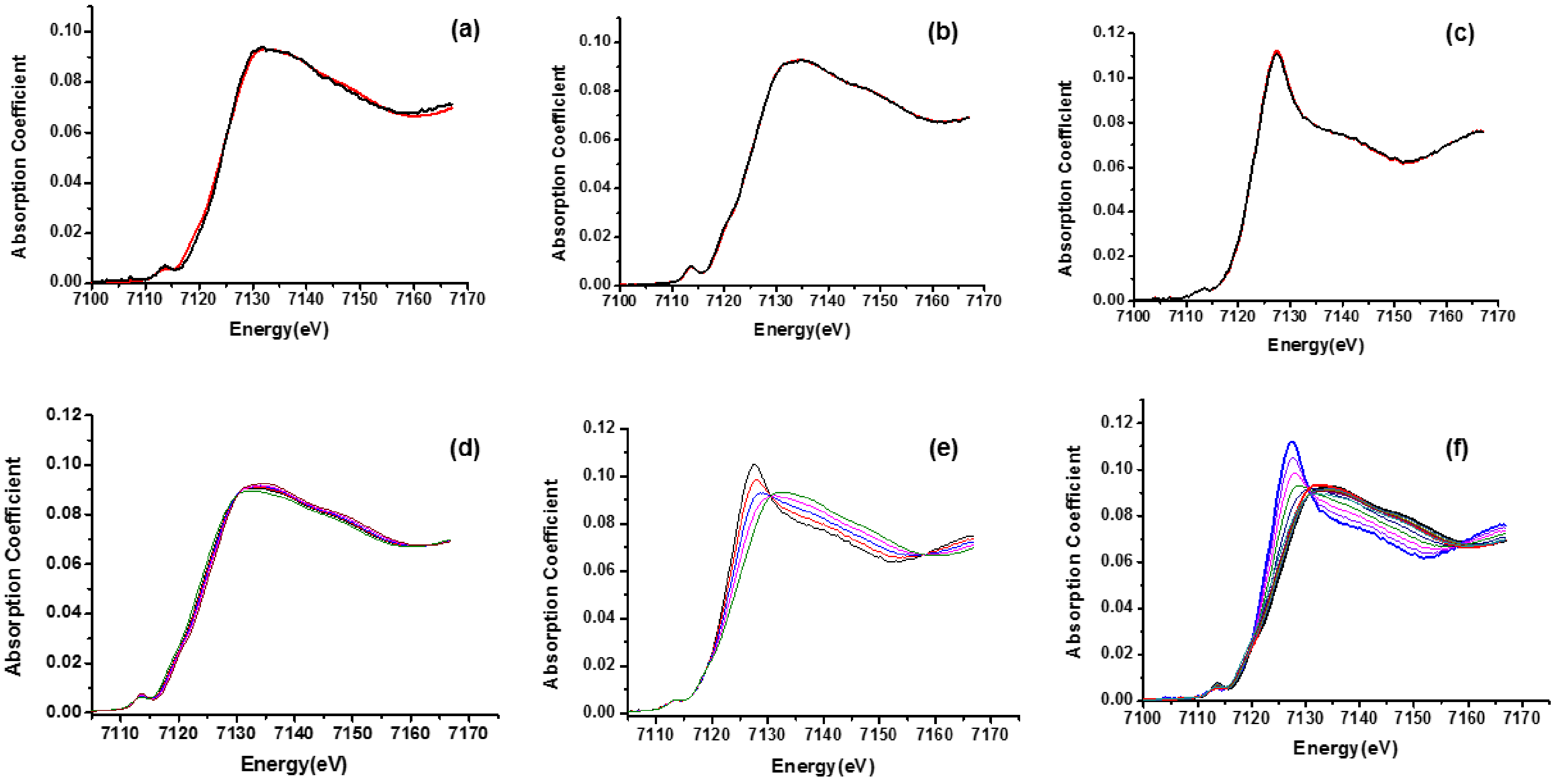

(a)
(b)
(c)
(d)
(e)
(f)
Absorption Coefficient
Energy(eV)

**Figure S3.** Interaction between AlkB and its redox partner, AlkG. (a) XAS profile for the fully oxidized AlkB and AlkG mixture (1:1) prior to the addition of dithionite (black) and the resulting profile from the linear combination of $AlkB_{oxidized}$ and $AlkG_{oxidized}$ spectra (1:1) (red). (b) XAS profile for the AlkB and AlkG mixture (1:1) in (a) after the addition of dithionite to the presumable mid-point dithionite concentration (black) and the resulting XAS profile from the linear combination of $AlkB_{oxidized}$ and $AlkG_{reduced}$ spectra (1:1) (red). (c) XAS profile for the AlkB and AlkG mixture (1:1) in (a) after the addition of dithionite to the presumable saturated dithionite concentration (black) and the resulting XAS profile from the linear combination of $AlkB_{reduced}$ and $AlkG_{reduced}$ spectra (1:1) (red). (d) Linear combination of XAS spectra resulted from various proportion of AlkG, from $AlkG_{oxidized}$ to $AlkG_{reduced}$, in the presence of $AlkB_{oxidized}$ (AlkG:AlkB = 1:1). The ratios of $AlkG_{reduced}$**:**$AlkG_{oxidized}$ from high to low mid-point edge energy are 0:100 (black), 26:74 (magenta), 46:54 (blue), 60:40 (red), 74:26 (wine red), and 100:0 (green), respectively; leading to the first phase ($AlkB_{oxidized}AlkG_{oxidized}$ →$AlkB_{oxidized}AlkG_{reduced}$) titration spectra, which correspond to spectral change from (a) to (b). (e) Linear combination of XAS spectra resulted from various proportion of AlkB, from $AlkB_{oxidized}$ to $AlkB_{reduced}$, in the presence of $AlkG_{reduced}$ (AlkG:AlkB = 1:1, samples were prepared with excess dithionite). The ratios of $AlkB_{reduced}$**:**$AlkB_{oxidized}$ from high to low mid-point edge energy are 20:80 (green), 60:40 (magenta), 40:60 (blue), 80:20 (red) and 100:0 (black), respectively; leading to the second phase ($AlkB_{oxidized}AlkG_{reduced}$ →$AlkB_{reduced}AlkG_{reduced}$) titration spectra, which correspond spectral changes from (b) to (c). (f) A series of simulated spectra from $AlkB_{oxidized}AlkG_{oxidized}$ (black)→ $AlkB_{oxidized}AlkG_{reduced}$ (red) → $AlkB_{reduced}AlkG_{reduced}$ (blue), which are consistent with the experimentally determined titration spectra obtained from AlkG:AlkB = 1:1 shown in Figure 2(b).

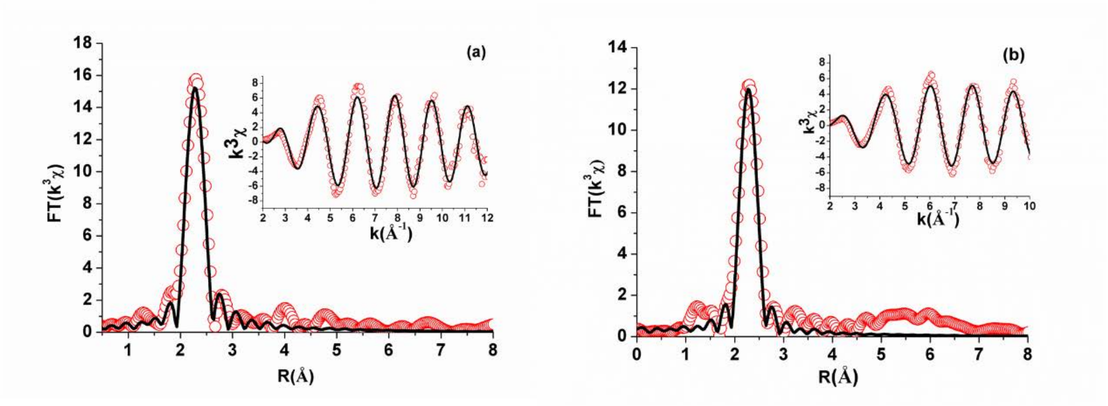


**Figure S4**. Fourier transforms of Fe EXAFS (red circles) and the corresponding best fits (black solid lines). Inset: Fe EXAFS (red circles) and the corresponding best fits (black solid lines). **(a)** Oxidized state (as isolated) of AlkG (rubredoxin-2); **(b)** reduced state of AlkG.

**Table S3.** Parameters used in the fitting of $k^3$-weighted EXAFS data for: **(a)** oxidized state (as isolated) AlkG (rubredoxin-2); **(b)** reduced state of AlkG in **Figure S4**. The error is estimated to be 25% for the coordination number and 0.01-0.03 Å for the distance. Parameters used in the fitting include: $N$, the coordination number; $R$ (Å), distance relative to Fe; and $\sigma^2$ (Å$^2$), the Debye-Waller factor; and $R_{fit}$ (%), the goodness-of-fit parameter. Fitting ranges for $k$ space (Å$^{-1}$) and $R$ space (Å) are indicated by $\Delta k$ and $\Delta R$, respectively.

| | **$AlkG_{oxidized}$** | | | **$AlkG_{reduced}$** | | |
|---|---|---|---|---|---|---|
| Bond Type | $N$ | $R$ (Å) | $\sigma^2$ (Å$^2$) | $N$ | $R$ (Å) | $\sigma^2$ (Å$^2$) |
| Fe-S | 4 | 2.25 | 0.002 | 4 | 2.28 | 0.003 |
| $\Delta k$ (Å$^{-1}$) | [2.14, 11.32] | | | [1.93, 11.22] | | |
| $\Delta R$ (Å) | [1.60 , 2.69] | | | [1.69 , 3.01] | | |
| $R_{fit}$ | 1.29% | | | 1.49% | | |

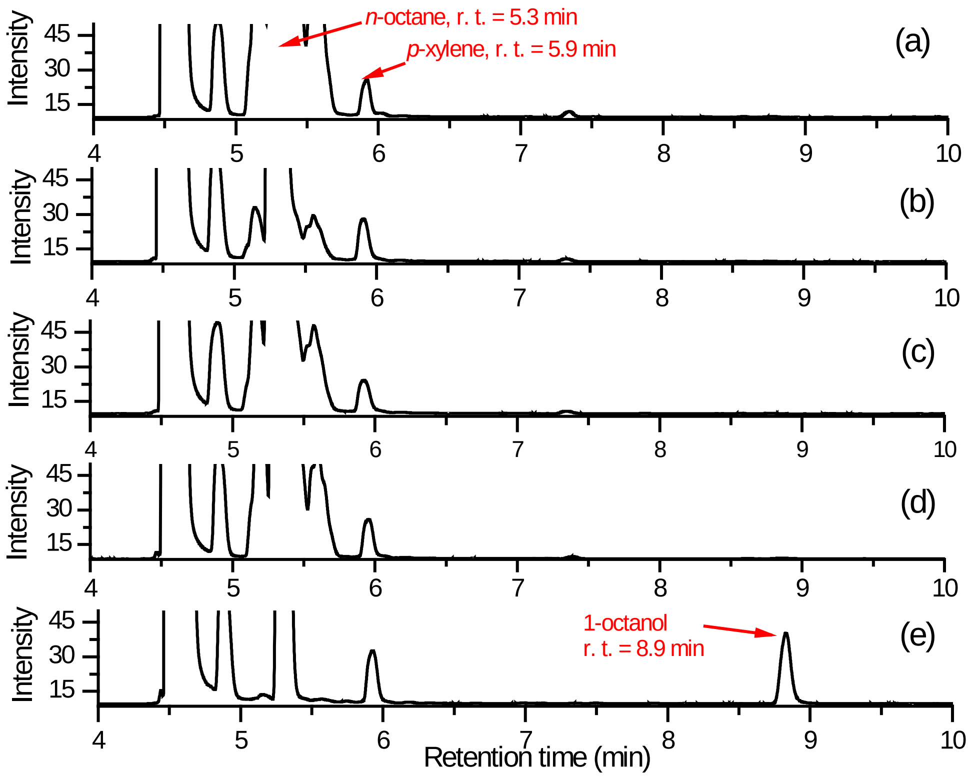


**Figure S5.** Electrochemical conversion of *n*-octane to 1-octanol analyzed by gas chromatography, from top to bottom: **(a)** blank electrode in (450 μL buffer + 50 μL *n*-octane) with no current applied, react for 30 min; **(b)** blank electrode in (450 μL buffer + 50 μL *n*-octane) with current applied, react for 30 min; **(c)** AlkG-immobilized electrode in (450 μL buffer + 50 μL *n*-octane), react for 30 min; **(d)** blank electrode in (450 μL AlkB-enriched membrane + 50 μL *n*-octane), react for 30 min; **(e)** AlkG-immobilized electrode in (450 μL AlkB-enriched membrane + 50 μL *n*-octane), react for 30 min. The retention time for *p*-xylene (internal standard) and 1-octanol are at 5.9 min and 8.9 min, respectively. The early eluted peaks come from the solvent used for extraction, dichloromethane (DCM), and other extractable components in the buffer mixture.

**Figure S6.** Electrochemical conversion of alkane to primary alcohol analyzed by gas chromatography. Top panel: GC chromatogram for the authentic standard of alkane substrate; middle panel: GC chromatogram for the authentic standard of primary alcohol product; bottom panel: GC chromatogram for the product obtained after the reaction mediated by AlkB on AlkG-immobilized SPCE. MTBE and *p*-xylene serve as the internal standards for the series of $C_3$–$C_4$ alkane/alcohol analysis and for the series of $C_5$–$C_{12}$ alkane/alcohol analysis, respectively. DCM: dichloromethane. MTBE: methyl *tert*-butyl ether (note: the retention time for chloroform in DCM resides at 4.9 min; the quantity of chloroform in DCM varies in different lots of DCM).

Propane:

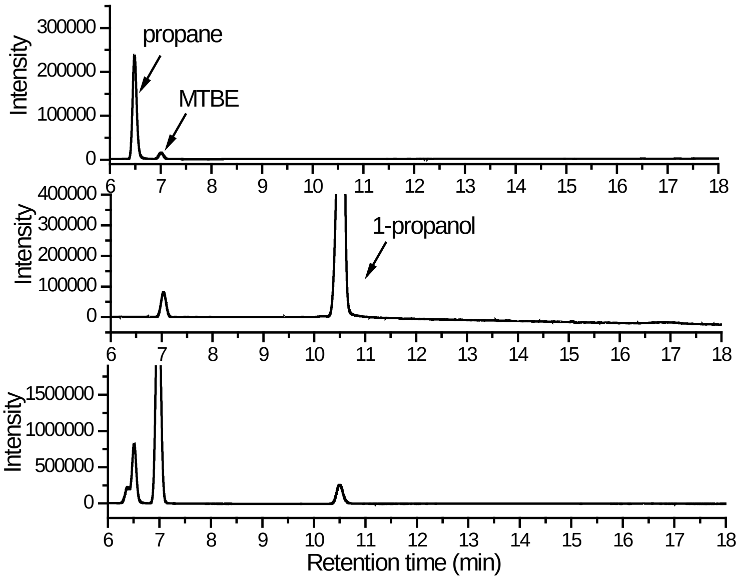


*n*-Butane:

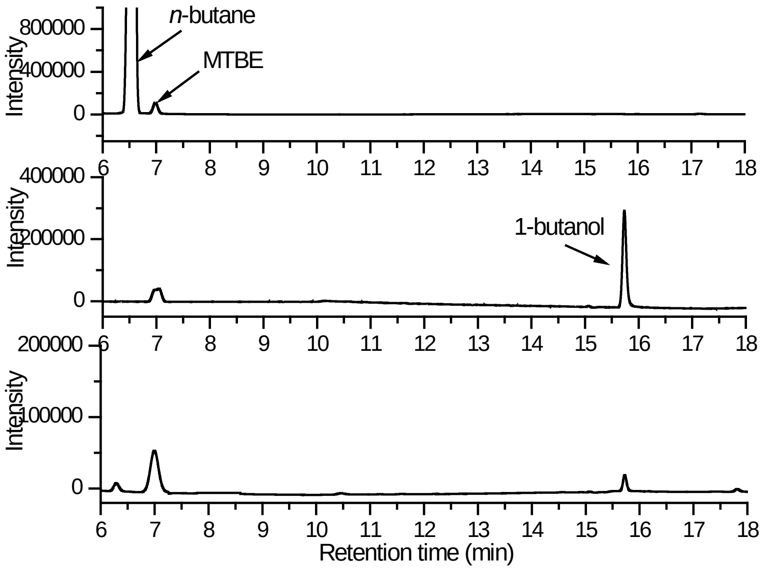

*n*-Pentane:

Note: In the bottom panel, the peak at 4.9 min is chloroform in DCM.

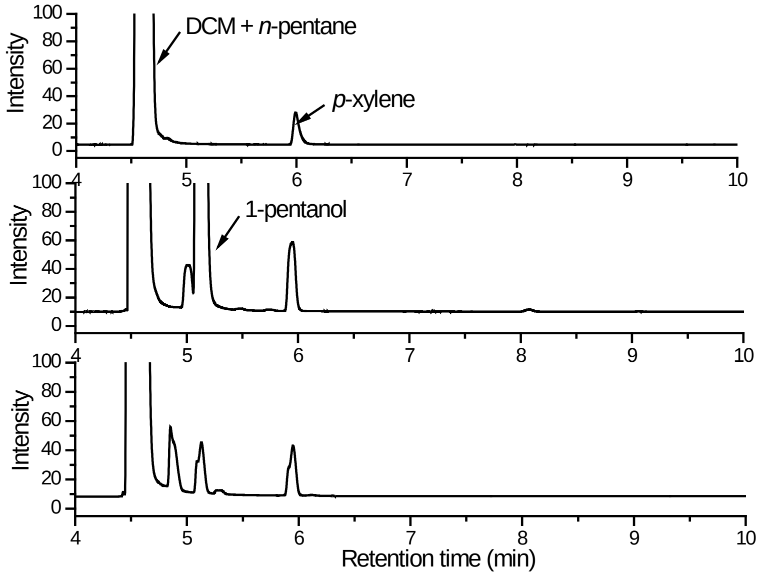


*n*-Hexane:

Note: In the bottom panel, the peak at 4.9 min is chloroform in DCM.

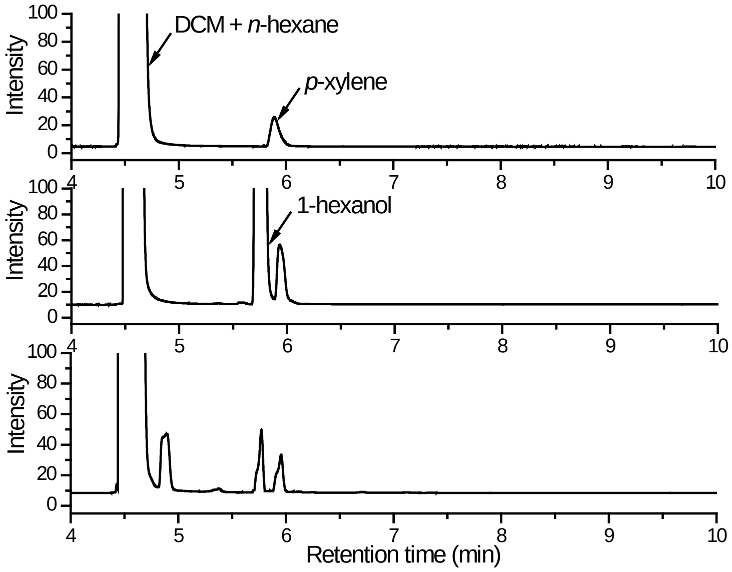

*n*-Heptane:

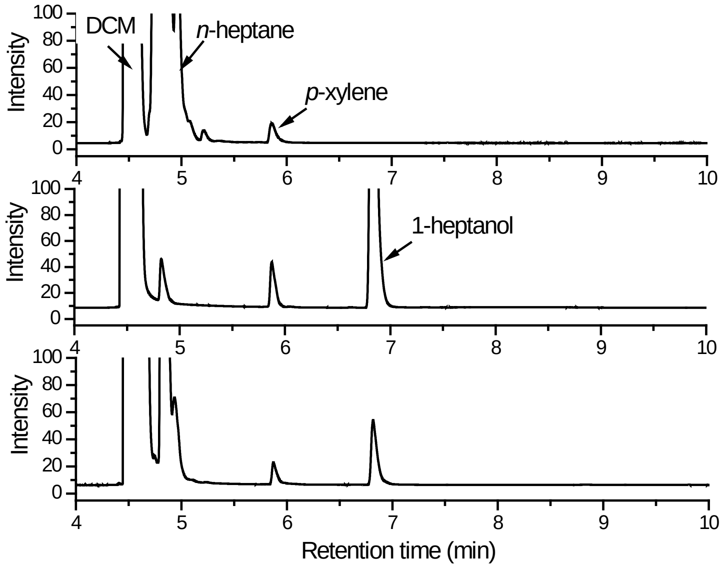


*n*-Octane:

Note: In the middle and bottom panels, the peak at 4.9 min is chloroform in DCM.

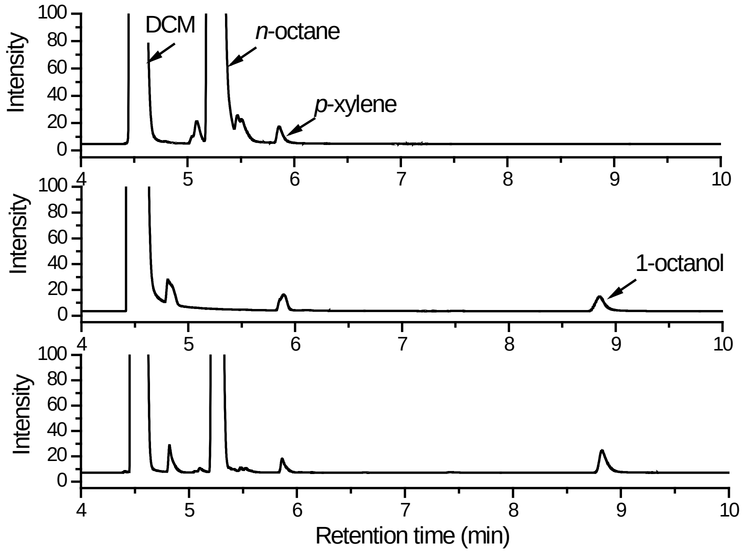

*n*-Nonane:

Note: In the middle panel, an extra peak in nonanol at 10.0 min is the impurity found in nonanol. In the bottom panel, the peak at 4.9 min is chloroform in DCM.

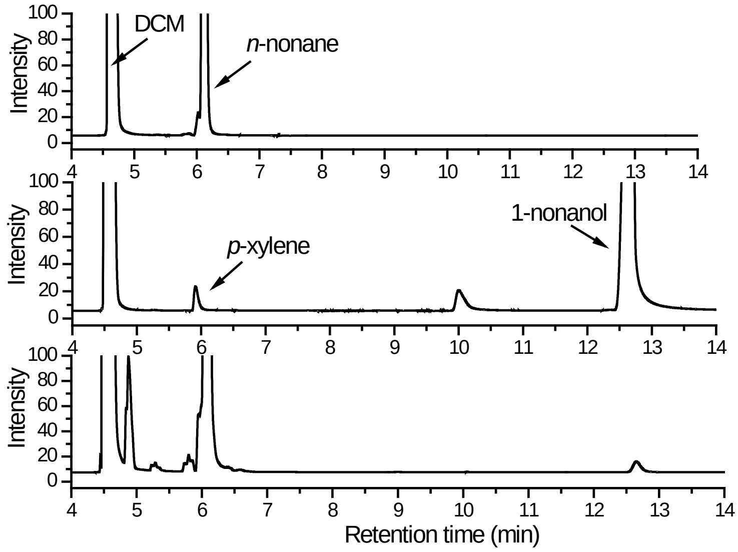


Decane:

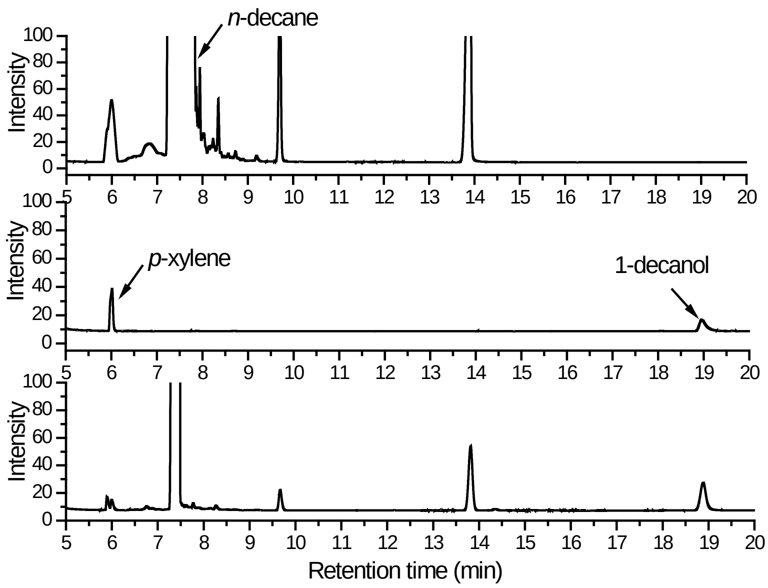

*n*-Undecane:

Note: An extra peak at 22.1 min is constantly found after the reaction. It originates from AlkB membrane and the identity is unclear. The quantity of this unidentified extractable substance varies after the extraction and has no particular relationship with the amount of product formation. Thus, this extractable matter is not involved in the reaction. In addition, its retention time is distinguishable from that of the primary alcohol product; thus, it does not interfere with the identification of product.

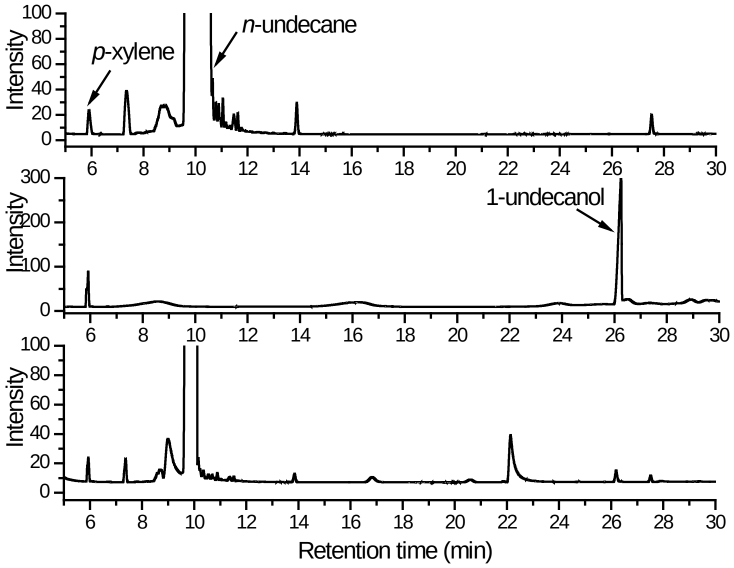


*n*-Dodecane:

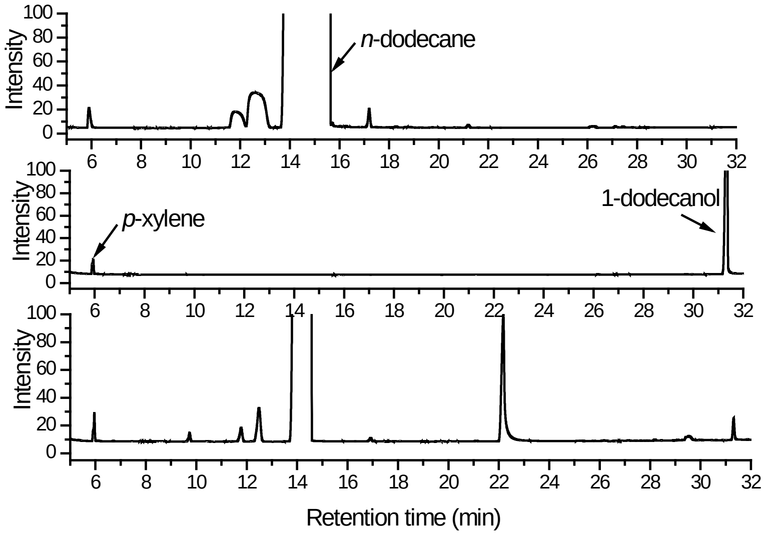

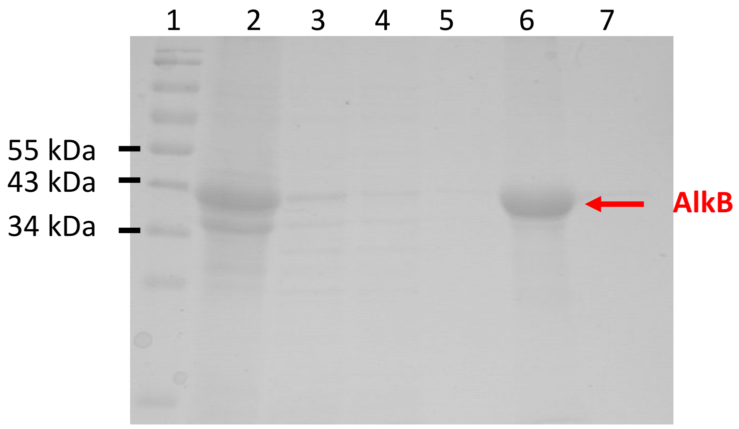


**Figure S7**. Purification of recombinant Strep II tagged AlkB using a StrepTactin™ column. AlkB is eluted by adding 0.5 mM D-desthiobiotin (DTBT) into the running buffer due to competitive-elution. Lane 1: MW marker; Lane 2: homogenate before column purification (7.5 μL); Lane 3: flow-through (7.5 μL); Lane 4: wash fraction 1 (7.5 μL); Lane 5: wash fraction 2 (7.5 μL); Lane 6: elution, with 0.5 mM DTBT (7.5 μL); Lane 7: elution, with 2.5 mM DTBT (7.5 μL).
